# Plasmonic–perovskite solar cells, light emitters, and sensors

**DOI:** 10.1038/s41378-021-00334-2

**Published:** 2022-01-12

**Authors:** Bin Ai, Ziwei Fan, Zi Jing Wong

**Affiliations:** 1 Department of Aerospace Engineering, Texas A&M University, College Station, TX 77843 USA; 2 School of Microelectronics and Communication Engineering, Chongqing University, 400044 Chongqing, P.R. China; 3 Chongqing Key Laboratory of Bioperception & Intelligent Information Processing, 400044 Chongqing, P.R. China; 4 Department of Materials Science and Engineering, Texas A&M University, College Station, TX 77843 USA

**Keywords:** Organic-inorganic nanostructures, Nanoparticles, Nanophotonics and plasmonics

## Abstract

The field of plasmonics explores the interaction between light and metallic micro/nanostructures and films. The collective oscillation of free electrons on metallic surfaces enables subwavelength optical confinement and enhanced light–matter interactions. In optoelectronics, perovskite materials are particularly attractive due to their excellent absorption, emission, and carrier transport properties, which lead to the improved performance of solar cells, light-emitting diodes (LEDs), lasers, photodetectors, and sensors. When perovskite materials are coupled with plasmonic structures, the device performance significantly improves owing to strong near-field and far-field optical enhancements, as well as the plasmoelectric effect. Here, we review recent theoretical and experimental works on plasmonic perovskite solar cells, light emitters, and sensors. The underlying physical mechanisms, design routes, device performances, and optimization strategies are summarized. This review also lays out challenges and future directions for the plasmonic perovskite research field toward next-generation optoelectronic technologies.

## Introduction

**Fig. 1:**
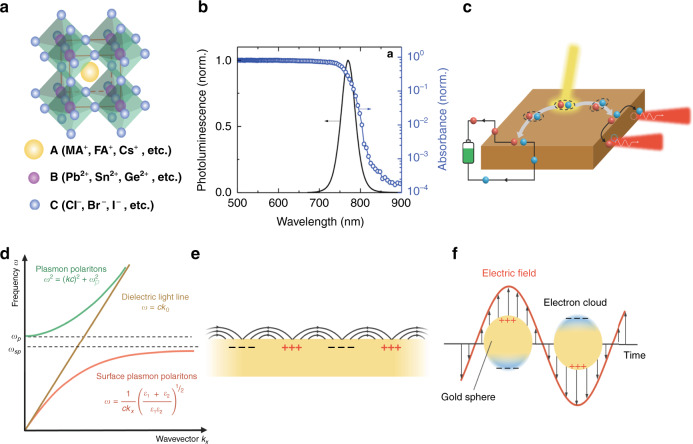
Outline of perovskites and plasmonics. **a** Structure of a halide perovskite unit cell. **b** Photoluminescence spectrum (left axis) and absorption spectrum as measured by photothermal deflection spectroscopy (PDS, right axis) of a (Cs_0.06_MA_0.15_FA_0.79_)Pb(I_0.85_Br_0.15_)_3_ thin film. Adapted with permission^213^. Copyright 2019, Wiley. **c** Schematic of the process of photocarrier extraction and light emission. **d** Photon dispersion in the bulk of a metal and surface plasmon polariton dispersion on the surface of the same metal and a dielectric. **e** Surface plasmon polariton (SPP) and (**f**) localized surface plasmon resonance modes (LSPRs).

Halide perovskite materials have an ABX_3_ chemical formula where A can be an organic or an inorganic cation (e.g., methylammonium (MA, CH_3_NH_3_^+^), formamidinium (FA, CH(NH_2_)_2_^+^), Cs^+^, Rb^+^, or their mixtures), B is a divalent cation (e.g., Pb^2+^ or Sn^2+^) and X is a halide anion (e.g., I^−^, Br^−^, Cl^−^ or their combinations). The lattice arrangement of perovskite is shown in Fig. 1a, where the larger atom A sits at the center of a cube, B occupies the corners of the cube, and the smaller atom X is on the faces of the cube. Halide perovskites have low recombination losses, large bandgap tunability, large absorption coefficients, high defect tolerance, and long charge carrier diffusion lengths and lifetimes^1–3^, all of which lead to efficient absorption, photocarrier extraction, and light emission properties, as shown in Fig. 1b, c. Moreover, halide perovskites can be solution processed at low temperatures, which significantly reduces their fabrication cost and complexity^4^. These advantages have led to the emergence of a variety of novel perovskite-based devices in the past decade^5,6^, such as solar cells (SCs)^7–10^, light-emitting diodes (LEDs)^11–14^, lasers^15–17^, photodetectors (PDs)^18–20^, sensors^21,22^, catalyst electrodes^23–25^, field-effect transistors^26,27^, fuel cells^28,29^, memory^30,31^ and spintronic devices^32,33^. However, halide perovskites are prone to phase changes and compositional degradation in the ambient environment. Despite that, the merits and prospects of halide perovskites are still very promising, and there are many passivation, encapsulation, compositional engineering, and novel deposition techniques to enhance halide perovskite stability.

The most prominent application of halide perovskites is as light-absorbing materials in solar cells. Miyasaka and coworkers first applied halide perovskite materials in dye-sensitized solar cells in 2009^34^. In 2012, Nam Gyu Park’s group revealed the great potential of perovskites by reporting a lead iodide perovskite solar cell (PSC) with a power conversion efficiency (PCE) above 9%^35^. Since then, PSCs have experienced rapid and continuous breakthroughs in regard to their PCEs. The larger bandgaps of halide perovskites compared with traditional photovoltaic materials such as Si and GaAs also enable them to form tandem solar cells with lower-bandgap photovoltaic materials to attain even higher PCEs. Furthermore, PSCs offer additional attributes such as their semitransparency, light weight, and flexibility. The PCEs of PSCs have now exceeded 25%, which is only slightly lower than the 27.6% PCE of the best single-crystalline silicon solar cells^36^. In addition to improving material quality and stability of perovskites, new design strategies to further improve the PCE are critical for next-generation photovoltaics (PVs).

Halide perovskites have also been used as light-emitting materials in LEDs and lasers. In perovskites, holes and electrons are confined into inorganic [PbX_6_]^4−^ octahedral networks, leading to strong Coulomb interactions and excitonic effects. They also have the advantages of a high quantum yield (QY), narrow band emission, and wide color tunability across the entire visible and infrared region, which makes them a promising material to use in light-emitting devices. CsPbBr_3_ perovskite nanocrystals (NCs) exhibit a high photoluminescence quantum yield (PLQY) of ~97%^37^. Moreover, the emission color can be facilely tuned in the visible range (blue to red) by varying the halide anion (Cl^−^, Br^−^, or I^−^), showing the potential of perovskites in white LEDs^38,39^. Recently, the efficiency of LEDs reached 108 cd/A (external quantum efficiency (EQE) of 23.4%) and was further increased to 205 cd/A (EQE of 45.5%) with a hemispherical lens^40^. Compared with those of mature technologies such as organic LEDs (EQE of 25%)^41^ and inorganic quantum dot LEDs (20.5%), this is a high efficiency^42^. Similarly, low-threshold lasers can also be achieved using halide perovskites as the gain materials. The optical gain coefficients of lead-halide perovskites can be comparable to those of conventional bulk semiconductors, such as GaAs^43^. Moreover, the emission wavelengths of lead-halide perovskite lasers can be tuned from ultraviolet to near-infrared (NIR) by controlling the composition^44–46^, filling the green emission gap between III-nitrides and III-phosphides. Various perovskite microlasers have been realized by microplates^47^, micro/nanorods^48^, microdisks^49^, and photonic crystals^50,51^. Recently, continuous wave (CW)-pumped MAPbX_3_ perovskites have been reported, showing the possibility of electrically driven perovskite microlasers^52^. Perovskite lasers can also realize unidirectional emissions^49^, mode control^53^, and high-density laser arrays^54,55^. Despite rapid progress, there is considerable room to improve the crystal quality and optical gain, as well as the EQE, lasing threshold, and emission lifetime.

Benefiting from the strong light absorption of halide perovskites, new applications have been found in regard to sensing and signal detection^56–60^. Novel perovskite detectors and sensors exhibit broad detection ranges, low detection limits, and fast response speeds. Xia et al. first reported a halide perovskite (CH_3_NH_3_PbI_3_) photodetector in 2014^61^. The responsivity and response time were 0.49 μA W^−1^ and 0.02 s, respectively. Four years later, halide perovskite PDs achieved a responsivity of 5.6 × 10^8^ A W^−1^, a detectivity of 2.8 × 10^16^ Jones, and a linear dynamic range of 92 dB^62^. Notably, every figure of merit is comparable with conventional complementary metal oxide semiconductor technologies; however, perovskites hold the advantages of a lower material cost and potentially greater flexibility. Halide perovskites have been used for different sensing applications, such as detecting volatile organic compounds^63^, liquid/solid compounds^64^, pH values/temperatures^65^, and pressures^66^. As researchers continue to expand the applicability of perovskite sensors, there is also a need to develop more compact sensing interfaces with a stronger light detection capability.

In the past decade, perovskite optoelectronic studies have mostly focused on enhancing the intrinsic properties of perovskite materials (e.g., via crystal quality optimization^7^, composition optimization^9^, and surface passivation^67,68^). However, there is always a ceiling for intrinsic property improvements, which calls for a new strategy to boost the performance of perovskite devices beyond the intrinsic limits of perovskites. This alternative strategy could be plasmonics, which can both enhance light absorption and modify electronic behavior^69–72^. The field of plasmonics relies on the collective oscillations of electrons excited by electromagnetic radiation at a metal–dielectric interface, which gives rise to the term surface plasmon resonance (SPR). SPR enables large wavevectors and thus field confinement (Fig. 1d)^73–75^. There are two types of plasmonic modes, propagating surface plasmon polaritons (SPPs) (Fig. 1e) and localized surface plasmon resonances (LSPRs) (Fig. 1f), and these are usually excited on metal films and metal nanoparticles (NPs), respectively. Light can be confined to the nanometer-deep subwavelength level in the proximity of a plasmonic nanostructure and induces strong electric field (*E*-field) enhancement. In addition to near-field enhancement, plasmonics can also result in strong far-field scattering, excitation of hot electrons, and localized photothermal heating. These effects are highly dependent on the structural morphology of metals, the permittivity of the surrounding dielectrics, and their light polarization and wavelength. In conventional optoelectronics (without perovskites), plasmonic effects have been intensively studied and proven to be effective. The ability of plasmonics to concentrate light at the nanoscale and enhance light–matter interaction holds the key to significantly improving the performance of perovskite optoelectronic devices.

Here, we review the recent works of plasmonic and perovskite-based solar cells, LEDs, lasers, sensors and other applications to provide a more comprehensive summary of plasmonic implementation and effects on halide perovskite devices. Both theoretical and experimental efforts will be covered, highlighting conceptual advances and key breakthroughs. The outline is as follows.

The first section (Plasmonic–perovskite solar cells) classifies the studies in plasmonic PSCs into NP-assisted and plasmonic film-assisted PSCs according to the structure of metal additives and the plasmonic modes. As NP-assisted PSCs have been widely studied both theoretically and experimentally, they are split into two separate subsections of simulation and experimentation to clearly distinguish between the theoretical predictions and attained experimental results. The effect of the configuration, geometry, size, and concentration of plasmonic NPs on solar cell performance is discussed. The second section (Plasmonic–perovskite light emitters) is focused on exploiting the plasmonic nanostructures in perovskite light emitters, including enhanced spontaneous emission and lasers. The third section (Plasmonic–perovskite sensors) discusses how plasmonics and perovskites are combined for sensors such as PDs and optical sensors. Other representative plasmonic–perovskite applications are presented in the fourth section. Regarding plasmonic–perovskite emitters, sensors, and other applications, specific simulation studies are rarer; thus, we introduce them together with experimental works. Finally, we summarize future avenues and challenges for plasmonic–perovskite applications.

## Plasmonic–perovskite solar cells

Plasmonic effects in SCs can be invoked by LSPRs and SPPs. LSPRs are mainly found in metal NPs and are dependent on the size, shape, material, and surrounding environment of the metal NPs. LSPRs lead to enhanced local *E*-fields and optical extinction. Maximum field enhancement is usually found at regions closest to the NP surface, and the field decreases exponentially within ~20–30 nm away from the surface. On the other hand, SPPs are excited by coupling light waves with electron oscillations on metal films. Incident light can therefore be converted into LSPRs and/or SPPs at different layers within the PSCs to attain higher light-harvesting efficiency. Plasmonic PSCs will be discussed in the subsections of NPs and plasmonic films.

### NP-assisted perovskite solar cells

PSCs are typically composed of an electron transport layer (ETL, e.g., TiO_2_ or phenyl-C_61_-butyric acid methyl ester (PCBM)), an active layer (halide perovskite), and a hole transport layer (HTL, e.g., poly(3,4-ethylenedioxythiophene)-poly(styrenesulfonate) (PEDOT:PSS) or spiro-OMeTAD) sandwiched by a transparent electrode (fluorine-doped tin oxide (FTO) or indium-doped tin oxide (ITO)) and a metal electrode (Au, Ag, or Al). In principle, NPs can be incorporated in ETLs, perovskite layers, and HTLs to alter light absorption. It is imperative to systematically summarize PV performances and clarify the underlying enhancement mechanisms and effects of the NP geometry, size, concentration, position, and composition. In the following, we first introduce the advances in simulation and then the experimental results.

#### Simulations of NP-assisted perovskite solar cells

**Fig. 2:**
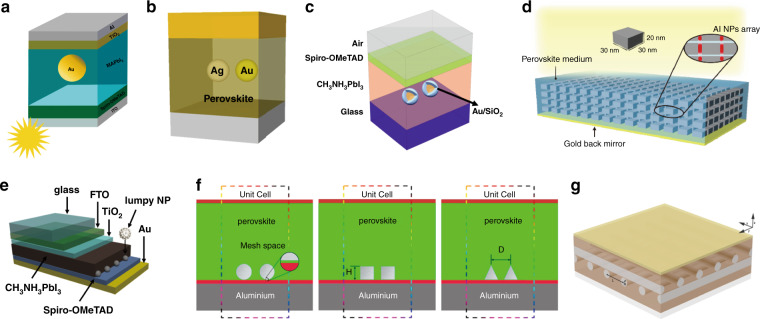
Different configurations of simulated plasmonic nanoparticle-assisted perovskite solar cells. Schematics of plasmonic–perovskite solar cells by incorporating (**a**) Au nanoparticles (NPs). Adapted with permission^76^. Copyright 2019, MPDI. **b** Ag–Au dimer. Adapted with permission^84^. Copyright 2017, SPIE. **c** Two Au–SiO_2_ core–shell nanoparticles. Adapted with permission^85^. Copyright 2018, IOP Publishing, Ltd. **d** Al nanoparticle array. Adapted with permission^87^. Copyright 2019, OSA Publishing. **e** Lumpy nanoparticles on the rear surface. Adapted with permission^90^. Copyright 2015, OSA Publishing. **f** Spherical, cylindrical, and conic NP arrays on the rear surfaces. Adapted with permission^91^. Copyright 2018, Elsevier. **g** AgNW nanocross array. Adapted with permission^93^. Copyright 2017, OSA Publishing. The TMM and 3D FDTD method were used for (**a**). The plasmon hybridization, dipolar–dipolar coupling method, and FDTD simulation were used for (**b**). The 3D FDTD method was used for (**c**). Effective medium theory and a detailed balance analysis were applied for (**d**). FDTD was used for (**e**–**g**).

Spherical NPs are used most commonly in PSCs and are usually studied by embedding them in the perovskite layer; one example is shown in Fig. 2a^76,77^. Chang et al. assessed the optical effects of Cu NPs embedded in a perovskite film by the transfer matrix method (TMM) and three-dimensional finite difference time domain (3D FDTD) method^78^. The thickness of the perovskite and the gap size between Cu NPs were varied, which led to the conclusions that Cu NPs could reduce the perovskite thickness from 400 nm to 300 nm while maintaining the absorption strength and that the absorbance at *λ* = 350–760 nm increased by 1.7% when the gap size was 30 nm (NP diameter was 70 nm). The trapping of incident light between the Cu NPs and perovskite led to a higher absorption efficiency. Palacios et al. presented an FDTD-based PSC model with Au NPs to analyze the effect of size, concentration, and location in the perovskite film^79^. Solar absorption could be enhanced by ∼10% when the thickness of the perovskite films was 200 nm and the radius of the spheres was 60 nm. The enhancement was ∼6% when the thickness of the perovskite films was 300 nm and the radius of the spheres was 90 nm. The enhanced absorption results arose from both the plasmonic near-field and scattering effects. Pathak et al. calculated the optical cross-section of arbitrarily sized and spherical-shaped metal NPs with perovskite by Mie scattering theory^80^. They found that NPs with a radius of 15 nm and volume concentration of approximately 10% achieved the highest scattering efficiency. The absorption and scattering efficiencies of Ag NPs with varying sizes, shapes, and polarizations of linearly polarized light were studied using the discrete dipole approximation (DDA) method in the active layer of PSCs^81^. Researchers found that the incorporation of Ag NPs in the active layer of PSCs led to a strong LSPR. Yue et al. proposed a full-wave simulation approach to demonstrate the effects of closely and regularly spaced Ag NPs in the perovskite layer of PSCs^82^. The infrared absorption of the PSC integrated with Ag NPs was enhanced by 58.2%. Coated and uncoated Ag NPs (radii ranging from 10 to 15 nm) were studied to understand the plasmonic interaction with perovskite dielectric media^83^. The SPR peaks of the coated Ag NPs could be tuned over a broader range than those of the uncoated Ag NPs.

In addition to works using isolated NPs (in the unit cell), the performance of dimers has been thoroughly studied. Hu et al. discussed the implication of employing random Ag–Au heterodimers in MAPbI_3_ SCs (Fig. 2b) by plasmon hybridization, the dipolar–dipolar coupling method, and FDTD simulation^84^. The Ag–Au heterodimers provided an enhanced optical field in both the junction (gap) and end areas (no-junction area near the surface of the NPs), while the Au/Ag homodimers only formed an enhanced optical field in the junction area. The absorption was enhanced by 28.15% for a 150-nm perovskite film embedded with random Ag–Au heterodimers (80-nm diameter and 25-nm gap) when compared to a perovskite film without NPs. The absorption enhancement was higher than that of Au homodimers whose absorption enhancement was 10%. The light absorption of the perovskite-embedded heterodimers was higher than that of the homodimers due to the radiation effect in the end area and the strongly enhanced local *E*-fields in the junction area. The performance of core–shell dimer NPs in PSCs was calculated by the 3D FDTD method (Fig. 2c)^85^. The maximum photocurrent of the Au–SiO_2_ core–shell dimer with a radius of 60 nm and a gap distance of 60 nm was 23.37 mA/cm^2^, which was larger than that of the Au dimer (22.5 mA/cm^2^), reference cell (19.8 mA/cm^2^), and cell with a single embedded NP (17.9 mA/cm^2^). The thin dielectric shell acted as an insulator to prevent charge recombination at the metal–absorber interface, leading to the largest enhancement in PSC performance.

Scaffolds were added to the perovskite for better dispersion of the NPs. Ghahremanirad et al. employed Au NPs in a kesterite mesostructure embedded in perovskite^86^. The plasmonic network and kesterite mesostructure can induce strong near fields in the absorber layer and enhance light absorption. As a result, more light will be confined within the perovskite layer. The absorbance spectrum was broadened, and the EQE of the planar PSC was enhanced by 29%. PSCs with Al NPs in the interstices of perovskite grids (Fig. 2d) were explored by effective medium theory and a detailed balance analysis^87^, showing that the performance of PSCs would be enhanced if the effective refractive index of the perovskites with Al NPs increased.

Several papers have modeled PSCs with plasmonic structures embedded in layers other than the perovskite layer. Hajjiah et al. studied the effect of adding Au and Ag NPs to the rear side of a PSC based on TMM^88^. The resonance wavelength of the NPs was tuned, especially in the wavelength range of red, to enhance the absorption of visible light. Both Au and Ag NPs led to a significant enhancement in the short current (*J*_sc_) when the size of the NPs exceeded 40 nm. Overall, the EQE was enhanced, and the EQE improvement was slightly higher with Ag NPs than with Au NPs. E. Ghahremanirad et al. theoretically demonstrated that the performance of planar PSCs was enhanced when HTL was a hexagonal NiO nanoprism array with Au NPs distributed in the gaps^89^. The calculations were based on the 3D FDTD method and the finite element method (FEM). The absorptivity of PSCs in the NIR region was enhanced by the Au NPs around the nanoprism array in the HTL. The Au NPs achieved the highest enhancement when the radius was 10 nm. The light-trapping ability of plasmonic nanostructures inside the active layer was increased by the tight coupling of the plasmonic NPs.

In addition to spherical NPs, NPs with various geometries have been examined in the search for better performance. Cai et al. studied the effects of adding lumpy Ag NPs to the rear facet of PSCs by running FDTD simulations (Fig. 2e)^90^. The lumpy NPs consisted of a large Ag core (radius = 100 nm) attached to small Ag NPs (radius = 10 nm). The lumpy Ag NPs provided the highest power conversion efficiency (PCE) when compared to the devices without NPs and with Ag (Au) spherical NPs. This enhancement was because the small Ag NPs enhanced the local *E*-field and the scattering was improved by the large Ag NPs over a broadband spectrum (300–900 nm). Pt NPs in triangular, rectangular, and spherical forms were embedded in the perovskite absorber (Fig. 2f)^91^. A finite-sized PSC model with different plasmonic arrays was constructed using the FDTD solutions of Maxwell’s equations. The triangular array showed almost the same performance as the rectangular array, decreasing the light reflectance to 6% and enhancing the current density by 22% from 39.2 mA/cm^2^ (without any other NPs) to 47.9 mA/cm^2^. Notably, the use of NPs in the active layer prevented other layers from parasitic absorption. Furthermore, cylinders^92^ and nanowires (NWs)^93^ (Fig. 2g) of different sizes, materials, and arrangements have also been studied and have achieved significant absorption and PCE enhancements.

Abdelraouf et al. calculated the effects of various nanostructured antireflective coatings, including spheres, cylinders, cones, cubes, and cylindrical shells, as well as their coupling, on the efficiency of PSCs based on Mie theory and a 3D FEM optical and electrical model^94^. It was found that the cones and cylindrical shells were not recommended due to the existence of various dipole modes that increased light losses in these nanostructures. The *J*_sc_ of PSCs employing the Ag spheres, cylinders, and cubes increased by 8.9%, 9%, and 3.5%, respectively. The enhancement of the TiO_2_ nanostructured coatings was larger than that of their Ag counterparts because TiO_2_ had a smaller absorption cross-section and a larger scattering cross-section over the solar spectrum. The *J*_sc_ of PSCs with TiO_2_ spheres and cylinders increased by 12.8% and 15%, respectively. Researchers also found that the metasurface cross-grating nanostructure within PSCs would increase the photocurrent and enhance the overall efficiency^95^. Enhanced light absorption and reduced light reflection are highly dependent on the dimensions, periodicity, and coating material of the grating nanostructures. TiO_2_ metasurfaces above and below the perovskite layer led to an increase in *J*_sc_ from 19.2 to 22.1 mA/cm^2^, while the Au metasurfaces achieved a *J*_sc_ of 21.13 mA/cm^2^.

More recently, Perrakis et al. presented detailed and systematic work on the implementation of metal NPs in PSCs^96^. CST Microwave Studio was used to perform 3D full-wave electromagnetic simulations. The PSC structure was SiO_2_ (1.1 mm)/ITO (100 nm)/PEDOT:PSS (40 nm)/MAPbI_3_/PCBM (50 nm)/Al (100 nm); the thickness of each layer is indicated in the brackets. Metal NPs with different materials, vertical positions, sizes, concentrations, clustering formations, and coatings were studied. It was demonstrated that (a) the optimum response of Ag NPs was with a radius of 60–70 nm, especially when in the proximity of the top of the perovskite layer. Furthermore, the optimum NP distance was ~300 nm; (b) the effect of NP randomness, angle of incidence, and polydispersity was very limited on the calculated enhancement; (c) the Al nanospheres with radii of 18 nm and a gap distance of 65 nm had the optimum performance; (d) the photocurrent was largely enhanced (up to 4.0% corresponding to photocurrent density (*J*_ph_) = 21.22 mA/cm^2^) when NPs were in the HTL (PEDOT:PSS, on top of the perovskite layer)and (e) the largest absorption enhancement was achieved when Al and Ag spheres were both used in the PEDOT:PSS layer and perovskite layer, respectively. Devices with Ag spheres embedded in the perovskite and Al spheres inside the HTL (PEDOT:PSS) can increase the PCE to ~12%. The origin of the photocurrent enhancement was attributed to the strong local *E*-field arising from the plasmonic resonances, and the larger scattering and antireflection due to the NPs, especially when the NPs were close to the top of the perovskite layer.

The above theoretical works studying the effect of plasmonic NPs on PSCs focused on characterizing PV performance, explaining the operational mechanism, and optimizing the NPs. Valuable primary results can be extracted from these theoretical models/simulations: (1) PV performance can be enhanced by incorporating NPs in PSCs; (2) the enhancement is due to the near-field absorption and far-field scattering of NPs induced by LSPR; (3) the geometry, size, material, density, distance, position, number, and coating all play important roles in enhancing PV performance. In addition, the absorption enhancement allows employment of PSCs with very thin perovskite layers, e.g., 150 nm, while at the same time maintaining the same performance as that of nonplasmonic PSCs with the optimum thickness (350 nm)^96^. Therefore, plasmonic NPs significantly reduce toxicity and make PSCs more environmentally friendly. The simulation demonstrates that embedding plasmonic NPs into PSCs is a powerful strategy to improve photoelectric performance. Although many simulations have been performed, challenges and issues still exist. The simulations should involve more materials and geometries of NPs. Most of the simulations are focused on NPs embedded in perovskite, while more works studying the effects of NPs in the ETLs, HTLs, and combined layers are needed to provide more design possibilities. There are nonnegligible differences between the simulation models and realistic devices, such as deviations in the optical constants of the materials and the film quality in the real device not being as perfect as that in the simulations. Most of the simulations are focused on optical absorption for evaluating PV performance. Furthermore, the electrical properties of the metal, which lead to an increase in conductivity and electron–hole recombination, are not taken into account. This will bring about large discrepancies between the simulations and real devices. Overall, the simulations provide important theoretical guidelines, but the experimental ways of implementing NPs in PSCs still need to be explored.

#### Experiments of NP-assisted perovskite solar cells

Generally, plasmonic PSC experiments are focused on the configuration (NP position), modification of the NPs (material, geometry, size, and concentration), PV performance (PCE) enhancement, and underlying mechanism. The results are summarized in Table 1 in chronological order. Statistical analysis is performed based on these results with the following conclusions.

**Table 1:** Embedded position, NP parameters (geometry, size, and concentration) PCE, and mechanisms of the experimental NP-assisted perovskite solar cells

Embedded position^a^	Geometry	Size (nm)^b^	Concentration (wt%)	PCE_ref_–PCE_NP_ PCE_relat_ (%)	Mechanism	Ref
m-Al_2_O_3_	Core–shell Au@SiO_2_ NPs	*D* = 80 *t* = 8	0.9	8.4–9.5 13.1	Reduced exciton binding energy with the incorporation of metal NPs, rather than enhanced light absorption	^193^
PEDOT	Ag nanotriangles	70	0.83	8.5–9.6 12.9	Increased plasmonic scattering effect	^194^
m-TiO_2_	Au-Ag alloy popcorn-shaped NPs	150 ± 50	0.7	8.9–10.3, 15.7	Optical absorption enhancement and faster charge transfer	^195^
m-Al_2_O_3_	Ag@TiO_2_	42	2.2	14.5–16.3 12.41	Light reemitted from the radiative recombination of electron–hole pairs and photon recycling	^115^
Spiro-OMeTAD–perovskite	Au NPs	15	0.01	12.66–12.74 0.63	LSPR and the electrical effect	^77^
m-TiO_2_	Au-decorated TiO_2_ nanofibers	58	0.3	9.23–14.92 61.64	Enhancement of light absorption, but also contributes to reduced charge recombination in Au@TiO_2_ nanofiber electrodes	^119^
m-TiO_2_	Ag NPs	30	20	4.57–6.15 34.57	Extends the optical pathway of incident light and the electron transport time	^196^
m-TiO_2_	Au@SiO_2_ nanorods	*L* = 39 *D* = 13 *t* = 15	4	12.4–14.4 13.5	Cross-sectional scattering and spectrally absorbed energy density.	^114^
Perovskite–PEDOT:PSS	Au@SiO_2_ nanorods	*L*= 34.7 *D* = 16.8 *t* = 9.5	0.047 pM	10.9–15.6 40	The LSPR of the Au@SiO_2_ nanorods could improve the incident light trapping as well as improve the transport and collection of the charge carrier	^101^
m-TiO_2_	Ag NPs	25	0.5	10.96–11.96 9.1	LSPR, charge trapping, and surface roughness	^107^
m-TiO_2_	SiO_2_@Ag@TiO_2_ NWs	10	N.A.^*c*^	12.17–15.09 24	Plasmonic-enhanced light absorption and increased recombination resistance	^197^
m-TiO_2_	Au nanostars	20–30	0.5	15.19–17.72 16.66	Enhanced light absorption and suppressed charge recombination	^108^
c-TiO_2_^*d*^	Au-Ag nanoalloy NPs	40	20	12.64–13.91 10.04	Increased light harvesting due to the increased optical path length caused by the light scattering of the metallic nanostructures	^110^
m-TiO_2_	Au@TiO_2_ nanorods	*L* = 32 *D* = 8 *t* = 5	1	15.51–16.78 8.2	Better charge separation/transfer as well as facilitated carrier transport in the presence of plasmonic NPs	^118^
m-TiO_2_	Au@Ag core–shell nanocuboids	*L*= 55 *W* = 11.5 *l* = 10–25 *t* = 6–10.5	2.9–3.8	15.16–18.31 20.78	Both plasmonic near-field enhancement and increased light-scattering effects	^116^
Spiro-OMeTAD	Au nanostars	35	0.02	12.49–13.97 14	SPR, backscattering, and charge transport	^121^
PCBM–BCP	Ag nanocubes	70	N.A.	11.9–13.3 11.76	Far-field scattering and optical near field	^139^
c-TiO_2_–FTO	Ag NPs	11	N.A.	7.78–7.08 22.5	Charge separation and trapping of electrons	^117^
m-TiO_2_ and perovskite	Au@TiO_2_	80	N.A.	12.59–18.24 44	Carrier transport	^102^
m-TiO_2_	Au@SiO_2_ core–shell NPs	20	1	3.78–4.49 18	Enhanced absorption	^126^
Perovskite–NiO	Au islands	10	N.A.	2.2–4.8 118.18	Strong scattering and enhancement of the *E*-field	^100^
PEDOT:PSS	Ag NPs SiO_2_ NPs	50	10^11^ particle mL^−1^	13.3–14 5.3	Increased charge selectivity and enhanced charge collection properties across the interface	^198^
Perovskite–TiO_2_	Ag@SiO_2_ NWs	*L* = 5–10 μm *D* = 80	0.06	14.32–18.03 25.9	Fast electron transmission, high light harvesting, electron–hole separation, and LSPR	^97^
m-TiO_2_	Au/Ag nanocubes capped with a thin ∼4 nm layer of SiO_2_	~ 60	1	3.9–4.9 26	Improved hot carrier thermalization to an equilibrium distribution, enhanced photoinduced carrier generation, and promoted efficient electron injection	^199^
m-TiO_2_	Au NPs coated by MgO	40	N.A.	12–16.1 34.2	Minimized photonic and energy losses for the generation of carriers, leading to a high charge transport capability and low charge recombination	^127^
m-TiO_2_	Au nanorod@SiO_2_	*L* = 40 *D* = 15 *t* = 8	0.09	14.39–17.39 20.85	Scattering effect, photon absorption, and superior enhanced charge separation	^200^
Perovskite–PEDOT:PSS	Au@SiO_2_ core–shell	*D* = 40 *t* = 2	1.2	11.44–14.57 27.3	LSPR, light absorption, faster charge transport time, and longer recombination lifetime	^99^
PCBM–perovskite	Ag NPs with propanethiol	N.A.	0.24	9.69–11.26 16.2	Scattering contributed to increased charge generation/transport with reduced recombination	^113^
m-VO_*x*_	Au nanobipyramids	*L* = 45–50 *D* =15–18	1.2	16.02–18.84 17.6	Stronger *E*-field enhancements and injection of hot holes	^201^
Perovskite–ZnO	Au nanorods	*L* = 50–70 *D* = 10	N.A.	16.51–14.47 11.61	Strong light absorption, hot electron transfer, reduced trap-state density, restrained charge recombination, and efficient electron transport	^98^
PCBM	Ag NPs	10.66 ± 1.8	5	7.34–11.90 60.76	Plasmonic-electrical effects	^120^
m-TiO_2_	Triangular-, rod-like-, and pentagonal-Au NPs with TiO_2_ shell	10–40 *t* = 10	0.01	15.04–17.85 18.7	Exhibit tunable LSPR wavelengths and functions as a “light tentacle” to improve the photoelectrical conversion efficiency	^202^
PEDOT:PSS	(AuAg) core–shell alloyed NCs	46	0.008	13.14–16.76 28	Better light harvesting by the scattering effect of the AuAg alloy NCs and better charge transport of the modified PEDOT:PSS layer	^111^
PCBM–perovskite	Crescent-shaped Ag NPs	79	N.A.	11.63–13.46 15.74	Near-field and far-field enhancement (plasmonic scattering) and a decrease in the hole injection barrier	^106^
Spiro-OMeTAD–perovskite	Cu−Ag alloy NPs	200	10% coverage	13.68–17.72 29.5	High electrical conductivity and absorption enhancement by effective light scattering	^104^
Spiro-OMeTAD–perovskite	Au NPs	30–35	N.A.	10.8–13.5 25	Enhanced charge separation and accelerated charge transfer due to hot electron generation in the Au NPs	^122^
m-TiO_2_ m-ZrO_2_	Au@Pt@Au core–shell NPs	18	1	12.4–13.4 8.06	Significant light trapping and utilization capabilities	^112^
m-TiO_2_	Au nanorod-NP dimers	*L* = 75 *W* = 18 *D* = 30	2.8	14.45–16.78 16	Broadband optical absorption enhancement	^109^
TiO_2_	Au NPs	16 ± 2	0.2	17.19–20.31 15.36	Improved light absorption, low charge recombination loss, enhanced carrier transport, and extraction with the plasmonic Au-TiO_2_/TiO_2_ dual ETL	^203^
SnO_2_	Au nanorods Upconversion NPs	N.A.	2	19.4–20.5 5.67	IR to visible upconversion luminescence and the scattering effect of the upconversion NPs	^204^
Spiro-OMeTAD–perovskite	Au@CdS nanospheres	*D* = 20 nm *t* = 15 nm	N.A.	17.71–21.38 20.72	Triggered heterogeneous nucleation of the perovskite precursor, lowered the valence band maximum, and localized surface plasmon resonance	^205^
Perovskite	Au NPs	15	N.A.	4.11–8.08 96.59	Enhanced light absorption capability and suppressed recombination rate of photogenerated electron–hole pairs	^206^
TiO_2_	Au NPs	12	N.A.	17.76–19.42 9.34	Hot electron transfer	^207^
Perovskite	Au/Cu_2_ZnSnS_4_ core/shell nanocrystals	10	N.A.	14.46–19.97 38.11	Reduction of recombination centers and increase of carrier lifetime	^208^
PCBM–Al electrode	Al NP–	20–70	N.A.	10.54–11.74 11.39	Improvement in the active layer due to photon absorption by both scattering and plasmonic effect in addition to reduced series resistance	^209^
Perovskite	Ag nanorods	*L* = 200 D = 20	N.A.	18.50–20.29 9.68	Localized surface plasmon resonance effect of the Ag NRs enhanced the light-capturing ability	^210^
PEDOT:PSS–perovskite	Au NP-decorated graphene oxides	28	N.A.	12.17–14.00 15.04	LSPR effects	^211^
TiO_2_	Au nanooctahedrons	Edge length = 115 nm	1 vol%	16.95–19.05 12.38	Enhanced light-trapping effect, reduced surface potential of the electron transport layer, and promoted effective photocarrier extraction at the interfaces	^212^

^a^ If the embedded position is in the form of *A*–*B*, *A* denotes the material coated on the NPs.

^b^ *L* is the length, *D* is the diameter, *W* is the width, and *t* is the thickness of shell.

^c^ Not addressed.

^d^ Compact TiO_2_.

The position of the NPs can be in the ETLs (PCBM, TiO_2_, Al_2_O_3_, and SnO_2_), perovskite, and HTLs (spiro-OMeTAD, PEDOT:PSS, and VO_*x*_). In simulations, perovskite is the major layer where the effect of NPs is studied, whereas the experiments mainly examine the use of plasmonic NPs in the ETLs and HTLs and at the ETL/HTL–perovskite interfaces. The NPs can be well blended into solutions of spiro-OMeTAD, PEDOT:PSS, and PCBM, which are then made into thin films as the building blocks of the device by facile spin-coating. Lee et al. found that when the spiro-OMeTAD HTL solution with Au NPs was spin-coated, the Au NPs could naturally stay close to the perovskite layer^77^. This rule likely also applies to the spin-coating of PEDOT:PSS and PCBM solutions with NPs. This distributes the NPs at the interface between the perovskite layer and spiro-OMeTAD or PBCM layer because these two materials are typically spin-coated after the perovskite layer. In contrast, the NPs prefer to stay far away from the perovskite in PEDOT:PSS, whose film formation is before the perovskite. The self-distribution of NPs by the spin-coating method deserves a more detailed investigation, as it can be a good way to control the NP position. The spin-coating of mixed solutions with NPs is also the major method to prepare NP-incorporated TiO_2_, Al_2_O_3_, and VO_*x*_ layers. They are mesoporous structures with a homogeneous distribution of NPs. NPs can also be incorporated in perovskites by spin-coating perovskite solutions blended with NPs^97^. However, in most cases, NPs are first deposited by spin-coating^98^, spraying^99^, or sputtering^100^, which are then coated by perovskite via spin-coating. Perovskite films with underlying NPs not only maintain their good quality but also show decreased roughness on the top perovskite surface and the absence of narrow gaps compared with perovskites without NPs^99,101^. It should be noted that one side of the NPs is attached on the surface of the TiO_2_, Al_2_O_3_, and VO_*x*_ scaffolds, while the other side is actually embedded in the perovskite, i.e., the NPs are at the perovskite–ETL/HTL interface. Analogously, for most other ETLs and HTLs, the NPs are at the perovskite–ETL/HTL interface. The position of the NPs is described by the form of the *A*–*B* interface in the first column in Table 1, where A denotes the material coated on the NPs.

**Fig. 3:**
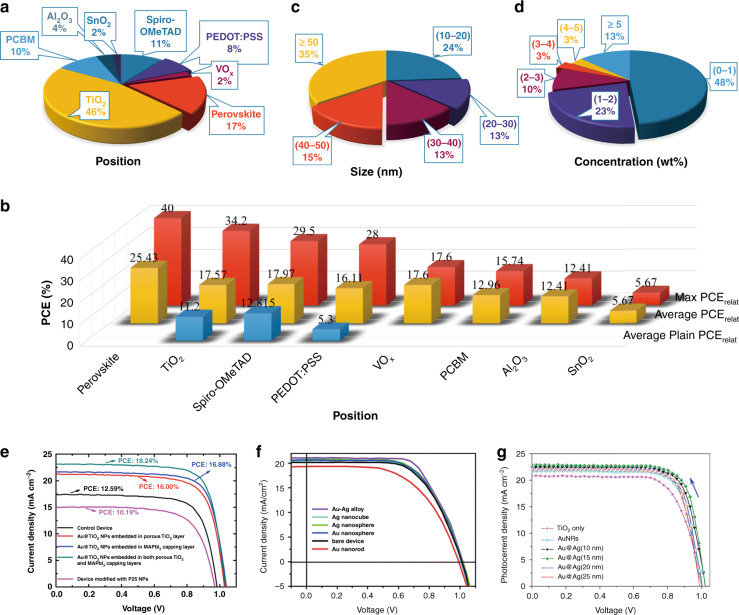
Summary of published experimental works on plasmonic–perovskite solar cells. **a** Breakdown of NP locations in perovskite solar cells. **b** Plots of max PCE_relat_, average PCE_relat_, and average plain PCE_relat_ as a function of NP locations. Distribution chart of (**c**) size and (**d**) concentration of NPs in perovskite solar cells. **e** *J*–*V* curves of perovskite solar cells with NPs embedded in different positions^102^. Copyright 2017, ACS. **f** *J*–*V* curves obtained for different types of metallic nanostructures^110^. Copyright 2017, RSC. **g** *J*–*V* curves of perovskite solar cells based on mesoscopic TiO_2_, AuNRs and different Au@Ag-incorporated electrodes^116^. Copyright 2017, Elsevier.

Figure 3a shows the ratio of the materials incorporated with NPs. The most commonly used material is TiO_2_ (22 in 48, i.e., 46% of the works), especially mesoporous TiO_2_ (m-TiO_2_, 17 works), followed by perovskites, spiro-OMeTAD, PCBM, PEDOT:PSS, Al_2_O_3_, VO_*x*_, and SnO_2_. The PCEs of the reference devices without NPs and the devices with NPs are denoted as PCE_ref_ and PCE_NP_, respectively. The relative enhancement is calculated as ${\rm{PCE}}_{{\rm{relat}}} = \frac{{{\rm{PCE}}_{{\rm{NP}}} - {\rm{PCE}}_{{\rm{ref}}}}}{{{\rm{PCE}}_{{\rm{ref}}}}} \times 100\%$. The PCE_ref_, PCE_NP_, and PCE_relat_ are listed in Table 1. For a fair comparison of the different incorporated materials, we exclude devices with PCE_ref_ < 10%. For the rest of the devices, the PCE_relat_ is analyzed by the maximum (max) PCE_relat_ (using any NPs), average plain PCE_relat_ (only using simple NPs with a spherical geometry and materials of Au or Ag), and the average PCE_relat_ (Fig. 3b). NPs incorporated in the perovskite layer show a maximum PCE_relat_ of 40%, while NPs incorporated in the TiO_2_ layer are slightly lower (34.2%). NPs incorporated in other material layers achieve lower efficiencies. Simple NPs are mainly incorporated in spiro-OMeTAD, PEDOT:PSS, and TiO_2_. The plain PCE_relat_ of spiro-OMeTAD is the highest, but in general, the plain PCE_relat_ is lower than the average PCE_relat_. The highest average PCE_relat_ (25.4%) is found in PSCs where NPs are located within the perovskite layer. This is followed by NPs embedded in spiro-OMeTAD, VO_*x*_, TiO_2_ and PEDOT:PSS, with an average PCE_relat_ between 16% and 18%. In comparison, NPs incorporated in PCBM and Al_2_O_3_ produce an inferior average PCE_relat_ in the 12% to 13% range, while NPs incorporated in SnO_2_ deliver the lowest average PCE_relat_ (below 6%). Overall, perovskite and TiO_2_ seem to be the best material layers for incorporating NPs to achieve a large PCE enhancement, especially when NPs are positioned close to the perovskite-m-TiO_2_ interface. Interestingly, Luo et al. incorporated Au@TiO_2_ NPs into both m-TiO_2_ and perovskite^102^ and obtained a maximum PCE_relat_ of 44% (Fig. 3e), which is the largest enhancement to the best of our knowledge. This result suggests that incorporating NPs in more than one position may achieve a higher efficiency, thus encouraging further exploration of multipositioned plasmonic NPs in PSCs.

The geometry of NPs varies in the form of spheres, nanostars, core–shell NPs, NWs, crescents, nanorods, nanocubes, triangles, pentagons, nanofibers, islands, nanobipyramids, popcorns, and nanorod dimers. NPs are either synthesized first by the seed-mediated growth method before incorporation or directly prepared in building blocks by using block copolymer micelle lithography (BCML)^103^, the wet chemical method^104^, the seed-mediated method^105^, thermal deposition^106^, and sputtering^100^. We classify the NPs into four types of structures: simple spherical NPs, core–shell NPs, shape-modified NPs, and dimers. Spherical Ag–NPs have been embedded into the TiO_2_ photoanode layer^107^, and the PCE of the PSC with Ag–NPs increased to 11.96% from 10.96%, which was a 9.1% enhancement. Li et al. integrated Au@TiO_2_ core–shell (@ means Au is the core and TiO_2_ is the shell) NPs into m-TiO_2_ and/or perovskite semiconductor capping layers^102^, enhancing the PCE from 12.59% (reference device without metal NPs) to 18.24% (Fig. 3e). Plasmonic Au nanostars have been incorporated into m-TiO_2_ for PSCs^108^, and the PSCs fabricated with TiO_2_–Au nanostars exhibited a PCE enhancement of 16.66%, increasing from 15.19% to 17.72%. Ma et al. showed that Au nanorod–NP dimers could enhance the PCE of PSCs by 16%^109^. The NPs of simple spherical NPs, core–shell NPs, shape-modified NPs, and dimers all brought about remarkable PCE enhancements. In particular, the latter three structures brought about higher PCEs than simple spherical NPs.

Materials such as Ag, Au, Au@SiO_2_, Ag@TiO_2_, SiO_2_@Ag@TiO_2_, Au@Ag, Au–Ag alloy, Au@Pt@Au, and Cu–Ag alloy were used to fabricate NPs. For instance, Au, Ag, and Au–Ag nanoalloy NPs with different sizes and shapes were embedded into the ETLs (TiO_2_) of PSCs via the physical deposition method^110^. The Au–Ag nanoalloy showed the best PCE improvement with a PCE = 14.8%, which was a PCE increase of 17.5% compared with bare PSCs (Fig. 3f). Sun et al. applied AuAg@AuAg (AuAg) core–shell alloy NCs (ANCs) into the PEDOT:PSS layers of PSCs as a dopant^111^. The highest PCE of the devices with AuAg ANC-doped PEDOT:PSS was 16.76%, which was an increase of 28% compared with that of the devices without AuAg ANCs (PCE = 13.14%). Chen et al. incorporated Cu–Ag alloy NPs to achieve a remarkable efficiency of 18.89%^104^. Au@Pt@Au core–shell NPs were incorporated into PSCs^112^, which increased the PCE by 8.1%, from 12.4% (normal PSCs without NPs) to 13.4%. It should be noted that NPs are usually coated by ligands (polyvinylpyrrolidone (PVP)) after the solution synthesis process^107^. Cho et al. replaced the original capping agent (PVP) with thermally stable propanethiol ligands by a ligand exchange method, which can prevent the aggregation of Ag NPs in the perovskite active layer^113^. The propanethiol ligands can improve the optical and morphological properties and lead to a better dispersion of the Ag NPs in the perovskite than that of the PVP-capped or bare Ag NPs. Regarding the core–shell structure, the shells can be SiO_2_^114^ and TiO_2_^115^. Tang et al. demonstrated that Au@SiO_2_ core–shell nanorods with optimized aspect ratios could greatly enhance the performance of MAPbI_3_ PSCs^114^. Such structures achieved a 16.1% improvement (from 12.4% to 14.4%) in the maximal EQE and increased the maximal *J*_sc_ by 13.5% from 20.0 to 22.7 mA/cm^2^. The open voltage (*V*_oc_) and filling factor (FF) remained almost the same. Core–shell Ag@TiO_2_ NPs were added to PSCs by a low-temperature processing route, enhancing the PCE up to 16.3%^115^. In general, the alloyed NPs show stronger enhancement.

Figure 3c, d shows the distribution of the sizes and concentrations of NPs that possess the largest PCE enhancement in each work. Approximately 90% of the works fall in the size range of 10–50 nm and concentration range of 0–5 wt%. Approximately half of the works use small NPs (<30 nm) and low concentrations (<1 wt%). Detailed results are presented to study the size effect. Au@Ag nanocuboids with lengths of 10, 15, 20, and 25 nm have been incorporated into an m-TiO_2_ layer, yielding PCEs of 16.89%, 17.21%, 16.35%, and 16.14%, respectively; these values correspond to relative enhancements of 11.4%, 13.5%, 7.8%, and 6.5% (Fig. 3g), respectively^116^. Au@Ag nanocuboids with a diameter of 15 nm show the best performance. Liu et al. investigated the effect of Ag NPs with diameters of 11 and 16 nm on the performance of PSCs^117^. PSCs with different NPs showed different *J*_sc_, *V*_oc_, and *FF* values. Overall, the NPs with a diameter of 11 nm showed the largest PCE. Regarding core–shell NPs, the thickness of the shell is important. Pathak et al. investigated the effect of the shell thickness by coating Au NRs with 5 and 10 nm TiO_2_ shells^118^. The performance of all devices with the Au@TiO_2_ nanorods (NRs) improved, regardless of the TiO_2_ shell thickness. However, their underlying mechanism for the performance enhancement was different. The *J*_sc_ of the devices with the Au@TiO_2_ NRs that had thicker shells remained the same, but their *V*_oc_ increased from 1.02 to 1.08 V compared with the devices without the NRs. In contrast, regarding the devices with thinner shells, *V*_oc_ remained unchanged, while *J*_sc_ exhibited an improvement from 20.68 to 22.23 mA/cm^2^. Particular efforts have also been devoted to the concentration effect. Malireport et al. studied the effect of 0.1 wt% to 0.5 wt% Au NPs in TiO_2_ on PSCs based on an in situ synthesis method of Au-embedded TiO_2_ nanofibers by an electrospinning technique^119^. The PCE, *V*_oc_, *J*_sc_, and *FF* increased as the NP concentration was increased from 0.1 wt% to 0.3 wt%; notably, these parameters decreased when the NP concentration was increased from 0.3 wt% to 0.5 wt%, i.e., the devices with 0.3 wt% attained the highest performance. Higgins et al. demonstrated planar PSCs with the structure ITO/PEDOT:PSS/MAPbI_3_/PCBM/Ni:Au, where PCBM was modified with variable amounts of Ag NPs (3, 5, 7, 10, 20, and 100 wt%)^120^. The addition of 5 wt% Ag NPs showed the largest PCE enhancement of 60.76%, increasing from 7.34% to 11.90%. Based on the above analysis, the size and concentration have different effects on the current, voltage, and *FF* and thus need to be optimized carefully to achieve the best performance.

**Fig. 4:**
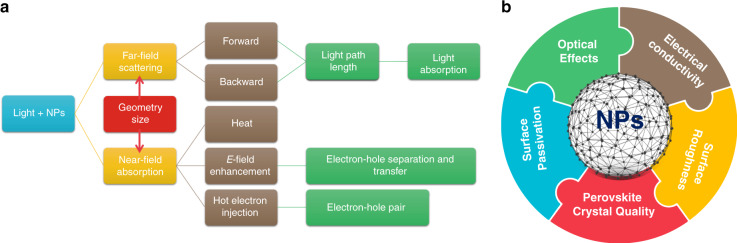
Mechanisms behind NP-assisted perovskite solar cells. **a** Far-field scattering and near-field absorption can both occur upon light illumination of NPs. Forward and backward far-field scattering will increase the optical path length, which leads to more absorption events within the light-absorbing semiconductor. Near-field absorption enhances the electric field, photothermal conversion, and hot electron injection. *E*-field enhancement brings stronger oscillation energy for faster electron–hole separation and electron (hole) transfer. Electrons will escape from the metal NPs to the active layer, inducing hot electron injection. This phenomenon provides extra electron–hole pairs for current extraction. **b** In addition to the optical effects, the incorporation of plasmonic nanoparticles in perovskite materials also influences the electrical conductivity, surface roughness, perovskite crystal quality, and surface passivation, which are all crucial factors to realize highly efficient and stable perovskite solar cells.

The underlying mechanism of plasmonic NPs in PSCs was discussed in previous reports. The keywords are presented in Table 1, including the LSPR, field enhancement, electron–hole separation, scattering, charge separation, light absorption, hot electron injection, and carrier transport. At times, the explanations are confusing and even conflicting. Here, we attempt to provide a comprehensive and consistent summary of the mechanisms by looking into the cause and effect, as shown in Fig. 4a. When light illuminates NPs, optical effects of near-field absorption and far-field scattering are generated. When the NPs are in front of the perovskite, i.e., light first reaches the NPs, which occurs for the PEDOT:PSS and TiO_2_ layers, forward scattering will increase the optical path length and enhance the light absorption of the perovskite, while backward scattering will produce a negative effect. Backward scattering will be beneficial if the NPs are placed beneath the perovskite (at the rear side closest to the metal electrode), for example, in the spiro-OMeTAD^121^ and PCBM^106^ layers. *J*_sc_ will improve due to enhanced light harvesting. The scattering effect depends on the size of the NPs. Large particles (>100 nm) are deemed more appropriate for scattering in PV applications^70^. However, the sizes of the NPs in 90% of the previous works are smaller than 50 nm (Fig. 3c). This indicates that the scattering effect of the NPs in the PSCs is minor. Near-field absorption leads to effects such as heat generation, *E*-field enhancement, and hot electron injection. As the light intensity is low, photothermal heating may be limited, which itself is interesting to verify experimentally. When NPs are in the perovskite, especially at the perovskite interface, the *E*-field enhancement brings about stronger oscillation energy for faster electron–hole separation and electron (hole) transfer, i.e., higher recombination resistance, resulting in a larger *FF*. Hot electron injection provides extra electron–hole pairs and thus leads to an improved *J*_sc_^118,122^. However, if the NPs are incorporated in the ETLs/HTLs and far away from the perovskite, the contribution of the near-field absorption effects is much reduced or even becomes negative. In addition to the light–NP interaction, other effects also take place (Fig. 4b). Metal NPs with dielectric shells improve the electrical conductivity, decrease the device resistance, and increase the *J*_sc_. In contrast, bare NPs and the agglomeration of NPs create charge recombination centers, which in turn degrade the overall PV performance. The surface roughness at the interfacial area between the carrier transport layer and perovskite layer increases due to the incorporation of NPs. The rough surface leads to a larger surface area and thus allows the collection of a larger number of photogenerated carriers^123^. The perovskite coverage can be increased by spin-coating PCBM with NPs because NPs can fill the empty spaces and passivate the grain boundaries^120^. NPs can also act as nucleation sites for perovskite films to improve the crystal quality^124,125^.

In summary, the PV performance of PSCs can be enhanced by plasmonic NPs because NPs lead to overall positive effects. The following conclusions are extracted based on the analysis of previous works: (1) The best position for using plasmonic NPs in PSCs is the perovskite-m-TiO_2_ interface because m-TiO_2_ is one of the best ETL materials that has good conductivity and a large surface area, and the plasmonic effects can both enhance the light–matter interaction of the perovskite and m-TiO_2_ layers. (2) NPs with core–shell structures, modified shapes, and dimers show stronger effects than simple spherical NPs. Bare NPs without any coating reduce PV performance (*V*_oc_) because they act as carrier recombination centers^117^. Hence, NPs are usually coated by ligands^113^, dielectric shells (SiO_2_ and TiO_2_)^126^, or additional dielectric layers^127^. Two resonance modes are excited along the long and short axes of the nanorods. This enhances the near-field absorption over a broader frequency bandwidth. The tips of NPs, such as triangles, cubes, pentagons, and popcorns, provide more hotspots than spherical NPs, leading to stronger LSPR and near-field absorption. NP dimers with small gaps have enhanced near-field absorption due to the stronger coupling between NPs. NPs with core–shell, dimer, and other modified shapes also show stronger effects than simple spherical NPs. (3) The alloyed NPs show a larger PV enhancement than that of a single material because the composite materials can broaden the absorption spectrum. (4) The size and concentration have different effects on the current, voltage, and *FF* and thus need to be optimized carefully. The size of the NPs should be tuned to make the absorption of NPs fall within the solar spectrum and overlap as much as possible, especially in the wavelength range where the intrinsic absorption of the perovskite is weak. Ultraviolet (UV) and NIR light can be absorbed by controlling the size of the NPs. In addition, the thickness of the shell is important. The interaction between the near-field absorption and perovskite can be changed due to the different shell thicknesses. When the shell is thick, the surface potential of the coating dielectric layer is changed by hot electron injection. As a result, the built-in potential of the device will increase^118^. The *V*_oc_ enhancement is not obvious once the shell is thinner than a certain threshold. In contrast, *J*_sc_ will be improved with a thinner dielectric layer because the interaction between the hot electrons from the metal and the excitons from the perovskite is strong and facilitates carrier separation and transport within the devices. A larger concentration of NPs results in stronger absorption; however, this also leads to agglomeration and recombination centers^119^. The majority of the NPs achieve a maximum PCE at a low concentration of <2 wt% (Fig. 3d). Based on the summarized results, we infer that alloyed core–shell nanorods (with sharp tips and sub-10 nm gaps) that are dispersed at the perovskite-m-TiO_2_ interface show promise for achieving higher PV performance. The sizes and concentrations may be optimal when <50 nm and <2 wt%, respectively.

### Plasmonic film-assisted perovskite solar cells

Plasmonic films are defined here as corrugated structures such as periodic arrays or random distributions of subwavelength holes (nanoholes) perforated in a metal film^128,129^ and structured continuous films^130,131^ that can excite the SPP mode or both the SPP and LSPR modes^132^. The plasmonic properties can be tuned by the film morphology, periodicity, and materials. The coupling between SPPs and LSPRs can result in unique optical effects and stronger light confinement. In addition, the penetration depth of SPPs into the dielectric can be 100 nm–1 μm, which is much larger than that of LSPR (<10 nm)^133^. This makes SPPs, in principle, able to exert a larger influence on the perovskite layer, ETL or HTL, leading to a higher PCE.

**Fig. 5:**
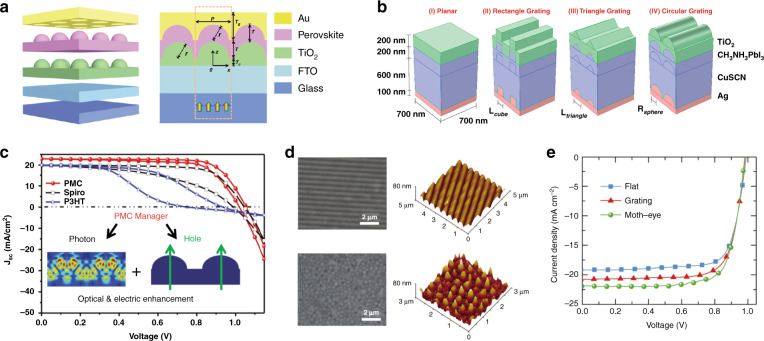
Plasmonic film-assisted perovskite solar cells. **a** 3D schematic diagrams and cross-sectional view of the hole-conductor-free PSC with the nanohemispherical Au electrode. Adapted with permission^135^. Copyright 2019, SPIE. **b** 3D schematics of the simulated PSC models with planar films, rectangular wire gratings, triangular wire gratings, and circular wire gratings. Adapted with permission^136^. Copyright 2018, Elsevier. **c** Performance comparison of PSCs with spiro-, P3HT, and periodic microstructure composite (PMC) HTLs. Adapted with permission^137^. Copyright 2016, RSC. The inset shows the simulated light field distribution of the 3D PMC HTL/Au at 668 nm and the enhanced optical transmission due to the hole. **d** SEM and AFM images of the grating and moth-eye patterned PCBM layers. **e** *J*–*V* curves of the flat, grating, and moth-eye patterned PSCs. Adapted with permission^138^. Copyright 2017, Wiley. The performance (*J*_sc_ and PCE) of the PSCs are improved by plasmonic films due to the great enhancement in light absorption, which is attributed to the excitation of the diverse plasmonic and photonic modes, including dipole-like LSPR, SPP modes, Bloch modes, cavity modes, and their mutual couplings.

Numerous simulations have been carried out to provide theoretical guidelines. Nanohemispherical Au electrodes have been designed for PSCs (Fig. 5a)^134,135^. The numerical results based on the FEM indicate that light absorption in these 2D photonic-structured PSCs is apparently stronger than that in a planar PSC. The integrated absorption efficiency reaches 65.7% over wavelengths ranging from 350 to 800 nm at normal incidence considering an air mass (AM) of 1.5 G solar irradiation, with an enhancement of 88.3% of that of the planar PSC. This great enhancement in light absorption is attributed to the excitation of the diverse plasmonic and photonic modes, including dipole-like LSPR, SPP modes, Bloch modes, cavity modes, and their mutual couplings. Abdelraouf et al. proposed a theoretical PSC model with front dielectric and back plasmonic wire gratings (Fig. 5b)^136^. The cross-sections of the grating with equally spaced wires were rectangular, triangular, and hemispherical. The light absorption was the highest for Ag rectangular wire gratings with side lengths of 110–150 nm, and *J*_sc_ increased by 22.4% compared with that of the planar structure. The *J*_sc_ of Ag triangular and semispherical wire gratings increased by 13.9% and 15.4%, respectively. The enhancement arose from both the contribution of the front dielectric wire grating and back plasmonic wire grating, with the former controlling the light-scattering directivity through Mie resonance and the latter solving the problem of low light absorption at longer wavelengths.

In experiments, Long et al. fabricated a periodic corrugated HTL and Au electrode^137^ (Fig. 5c). The absorption was stronger for the periodic microstructure composite HTL with the conformal Au electrode compared with the planar HTL and electrode due to enhanced cavity effects. Both Fabry–Pérot resonance and SPR played a role in the cavity, increasing the light-harvesting efficiency through the coupling between light and surface plasmons. Wei et al. fabricated a metal back electrode into a moth-eye nanostructure via a soft imprinting technique (Fig. 5d,e)^138^. Compared with the flat reference cell, the *J*_sc_ of the patterned devices with moth-eye nanostructures was enhanced by 14.3%. Its PCE was up to 16.31% without sacrificing the *V*_oc_ and FF. The moth-eye back electrode enhanced the light scattering over a broad frequency band and induced plasmonic effects for stronger light confinement in the device, leading to performance enhancements. The combination of metallic films and NPs has also been explored. Kim et al. developed planar PSCs with both plasmonic Ag nanocubes and Ag back electrode films^139^. An ETL was inserted between the nanocubes and electrode, whose thickness was varied to tune the coupling between the Ag nanocubes and the back electrode to make the plasmonic absorption peak close to the absorption edge of the perovskite layer. The far-field scattering and near-field absorption around the nanocube face closest to the perovskite layer were also greatly enhanced by the coupling. The average PCE was enhanced from 11.9% to 13.3% due to plasmonic coupling.

Plasmonic films have not been used as widely as NPs because the fabrication of corrugated plasmonic films in PSCs is usually more complex. In addition, the transmission of plasmonic films is low, so plasmonic films can only be used on the rear side of PSCs. Nevertheless, these pioneering works have shown the great potential of plasmonic films to increase the efficiency of PSCs.

## Plasmonic–perovskite light emitters

### Enhanced spontaneous emission

**Fig. 6:**
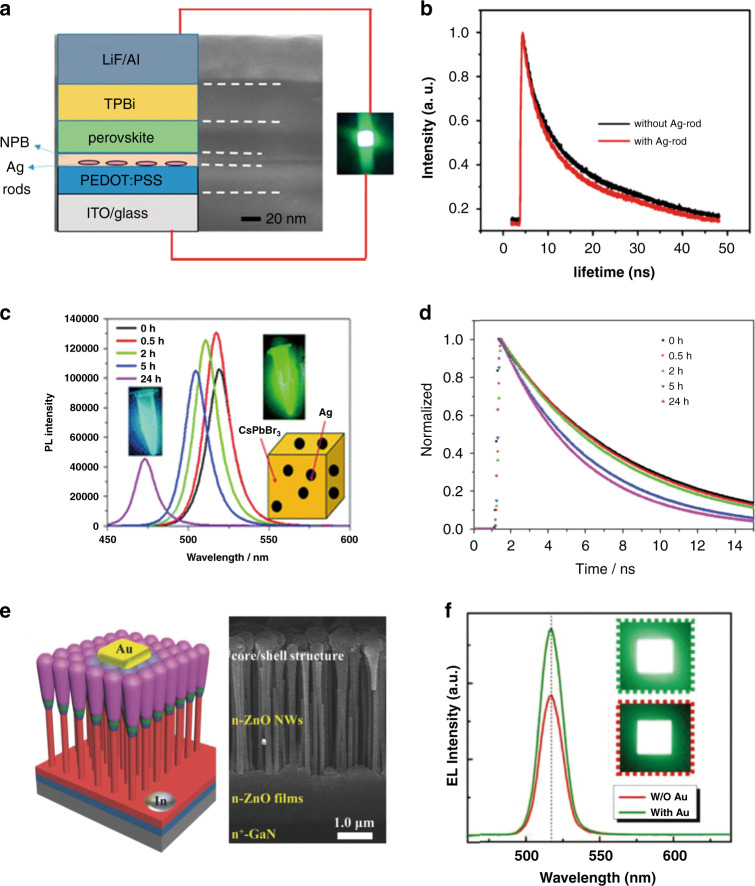
Plasmonic–perovskite light emitters with enhanced spontaneous emission. **a** TEM image of a cross-section, the corresponding schematic diagram, and the photographic image of the plasmonic–perovskite LED based on the Ag-CsPbBr_3_ system. Adapted with permission^140^. Copyright 2017, ACS. **b** PL lifetime of the perovskite CsPbBr_3_ nanocrystal layer with and without Ag rods. **c** PL intensity and (**d**) fitted time-resolved photoluminescence decay spectra of CsPbCl_x_Br_3−x_@Ag hybrid nanocrystals obtained by reacting CsPbBr_3_ nanocrystals with AgCl for 0–24 h. The inset in (**c**) shows photographs of the CsPbBr_3_ nanocrystals and CsPbCl_x_Br_3−*x*_@Ag hybrid nanocrystals at a reaction time of 24 h under 365 nm UV light irradiation. The enhancement is mainly attributed to the enhanced absorbance of UV or blue light by the Ag-induced plasmonic near-field effect. Adapted with permission^142^. Copyright 2017, RSC. **e** Left image: schematic of the Au/p-NiO/CsPbBr_3_ QD/MgZnO/Au NP/n-ZnO/n^+^-GaN heterostructure LED. Right image: cross-sectional SEM image of the NiO/CsPbBr_3_ QD/MgZnO/Au NP/ZnO coaxial NWs. (**f**) EL spectra of the plasmonic PeLED with Au NP decoration and the reference device without Au NPs captured at 8.0 V. The insets present the corresponding emission photographs of an emitting unit (2 × 2 mm^2^). The electroluminescence enhancement is associated with the increased spontaneous emission rate and improved internal QY induced by exciton–LSPR coupling. Adapted with permission^150^. Copyright 2018, Wiley.

Halide perovskite materials exhibit strong spontaneous emission, whose wavelengths can be tuned throughout the visible range by mixing halide anions. This makes perovskites a promising platform for creating efficient, low-cost and multicolor optoelectronic devices such as LEDs and displays. Incorporating plasmonic nanostructures into LEDs to induce coupling between excitons (i.e., electron–hole pairs bound by Coulombic interactions) and surface plasmons is a powerful strategy to enhance the performance of perovskite LEDs (Fig. 6a)^140^. In theory, if the energy of the excitons of perovskite matches the SP resonant energy, the two energies will couple and enhance the scattering or free-space re-emission. This leads to an extra recombination pathway, which significantly increases the spontaneous radiation rate (Fig. 6b) and improves emission performance.

The plasmonic effects on perovskite LEDs are studied mainly based on NPs. Mokkath et al. calculated the optical properties of Au-doped all-inorganic CsPbX_3_ (*X* = Cl, Br, I) perovskite quantum dots (QDs) based on time-dependent density functional theory^141^. Their results confirmed deep-level trap states induced by the Au dopant, a significant absorption redshift, and the emergence of *E*-field hotspot regions. CsPbBr_3_@Ag hybrid NCs were synthesized by reacting CsPbBr_3_ NCs with Ag*X* (*X* = Cl, Br, or I) powders in hexane^142^. Briefly, 2–5 nm Ag NPs were nucleated and attached randomly on the surface of CsPbBr_3_ NCs. The PL intensity and emission lifetime of the CsPbBr_3_@Ag hybrid NCs were significantly enhanced and reduced, respectively, compared with those of pure CsPbBr_3_ NCs when illuminated by 400 nm light (Fig. 6c, d). The enhancement was mainly attributed to the plasmonic effects of the Ag NPs enhancing the absorbance of UV or blue light. However, Ag adhesion deteriorated the surface quality of the CsPbBr_3_ NCs and in turn undermined the PLQY. Therefore, Ag adhesion has both positive and negative effects on the PL of hybrid perovskite QDs. To achieve an enhanced PL, the size and density of Ag NPs need to be optimized so that the positive effect is stronger than the negative effect. Xu et al. reported a perovskite blue LED with Au NPs embedded in PEDOT:PSS^143^. The fluorescence intensity was improved by 3.8 times. The maximum luminance and EQE reached ~1110 cd/m^2^ and 1.64%, respectively.

Liu et al. placed disordered Au NRs underneath a film of CsPbBr_3_ NCs^144^ and studied their emission performance. By using femtosecond laser pulses at 800 nm, amplified spontaneous emission (ASE) at approximately 523 nm was excited by two-photon pumping. A narrow peak with a linewidth of 5 nm appeared with the pump fluence reaching a low threshold of 0.65 mJ/cm^2^, owing to the plasmonic effects of Au NRs improving the emission transition rate. et al. precipitated Ag NPs and CsPbBr_3_ perovskite QDs in borosilicate glass by the conventional melt-quenching method^145^. Part of Pb^2+^ was replaced by Ag^+^ in the CsPbBr_3_ QDs, and the remaining Ag^+^, which did not react with Pb^2+^, was converted into Ag NPs. These Ag NPs enhanced the PL by 5 times for the CsPbBr_3_ QD-doped glasses, which was mainly due to the enhanced optical absorption and LSPR of the Ag NPs. Yun et al. proposed stretchable plasmonic templates of Au and Au/SiO_2_ NPs embedded in polymer to improve the luminescence of CsPbBr_3_ NCs^146^. The charge carrier imbalance due to hole trapping in the perovskite LEDs with naked NPs could be prevented by embedding Au or Au/SiO_2_ NPs in the polymer. The luminescence of the perovskite NC film on the template with Au/SiO_2_ NPs increased by 54% compared with that of the perovskite NC film on the reference (polymer film without metal NPs). The luminescence enhancement of Au/SiO_2_ NPs was ascribed to coupling to the LSPRs of the Au/SiO_2_ NPs.

Li et al. studied the performance of perovskite QD (PQD) films coupled to Ag NW networks (NWKs) with a spacer of polyvinyl alcohol (PVA) between the PQD film and NWKs^147^. Compared with bare quartz, the PVA substrate significantly enhanced the emission intensity but reduced the emission rate of PQD excitons. When NWK was introduced, the emission intensity and rate exhibited a maximum of 6-fold (average 3.4-fold) and 2.4-fold (average 1.9-fold) increases, respectively, due to efficient NWK–PQD coupling. They further demonstrated a Purcell-enhanced emission rate in a hybrid structure of PQDs with plasmonic crystals at room temperature^148^. Colloidal PQDs, Ag nanocubes, and PVP have been incorporated together into a device by chemical assembly. The PL enhancement can be modified in both the steady-state fluorescence and time-resolved measurements by changing the PVP spacer thickness and Ag nanocube surface density. The total fluorescence intensity and emission rate demonstrate 3.5-fold and 4.5-fold enhancements, respectively.

A plasmonic Au NR/CsPbBr_3_ QD film was fabricated via spin-coating^149^. The PL intensity of the Au NR/CsPbBr_3_ QD film demonstrated a 2-fold enhancement compared to that of the CsPbBr_3_ QD film without Au NRs. The CsPbBr_3_ QD film exhibited a lifetime decrease from 6.15 ns to 4.78 ns after incorporating Au nanorods. The Au nanorods triggered an LSPR of approximately 500 nm, which enhanced the emission intensity of the CsPbBr_3_ QD film. Shi et al. fabricated a Au/p-NiO/CsPbBr_3_ QD/MgZnO/Au NP/n-ZnO/n^+^-GaN heterostructure LED (Fig. 6e, f)^150^. The emission was enhanced by 1.55 times after embedding Au NPs into the device. The luminance, EQE, and current efficiency of the optimized plasmonic–perovskite LED reached 10206 cd/m^2^, ~4.626%, and 8.736 cd/A, respectively. The electroluminescence performance was enhanced due to the increased spontaneous emission rate and improved internal QY, which was induced by the coupling between the exciton and LSPR.

Plasmonic films can also enhance the PL. Chen et al. placed CsPbBr_3_ nanoplatelets between Ag NWs and a Ag film. Strong biexciton emission was induced with continuous-wave excitation at room temperature^151^. The exciton occupancy for generating biexcitons was reduced by ~10^6^ times in the Ag NW-Ag film nanogaps. The nonlinear Fano resonance between biexcitons and plasmonic cavity modes was responsible for the great enhancement in biexciton emissions. Adamo et al. coated MAPbI_3_ on a Au slit film^152^. The luminescence intensity was enhanced by more than 10 times compared with that of pure MAPbI_3_, while the luminescence lifetime demonstrated a nearly 3-fold decrease. The improvement in luminescence intensity was attributed to the significantly enhanced Purcell effect with an enhancement factor of more than 300.

Plasmonic NPs and films with Au and Ag materials have been used in perovskite LEDs and have achieved impressive PL and EQE enhancements. It is expected that more nanostructures and different material combinations with stronger plasmonic enhancement will be explored for perovskite LEDs. The impact of the plasmonic nanostructure on the crystal quality and stability should also be studied more thoroughly.

### Plasmonic–perovskite lasers

The properties of plasmonic-based perovskite lasers, such as the threshold, wavelength, polarization, and laser emission characteristics, can be facilely controlled by the rational design of the coupling between perovskites and plasmonic nanostructures. Plasmonic lasers with a low mode volume have been realized by coupling the perovskite with plasmonic NPs and films. This progress advances the development of high-performance perovskite micro/nanolasers.

**Fig. 7:**
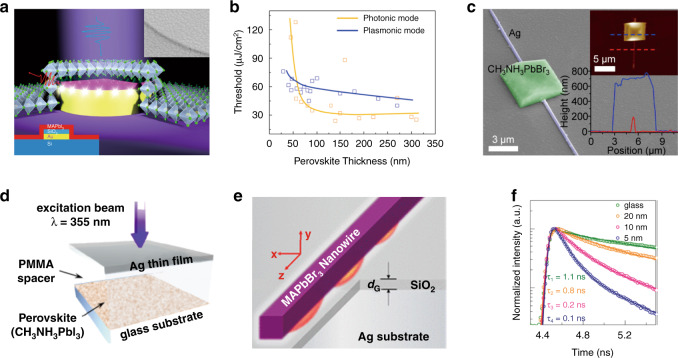
Plasmonic–perovskite lasers. **a** Schematic of a spaser (surface plasmon amplification by stimulated emission of radiation) composed of a MAPbI_3_ NP/SiO_2_/Ag plasmonic laser. Inset: SEM image of perovskite NPs lying on Au/SiO_2_ microdisks. In contrast to previous reports, here, the spasers are determined by the boundary of Au patterns instead of the crystal facets of MAPbX_3_ nanosheets. As a result, whispering gallery mode-based circular spasers and spaser arrays are successfully realized by patterning the Au substrate into circles and gratings, respectively. **b** Lasing threshold of plasmonic and photonic lasers versus perovskite thickness; the solid lines are used for guidance. Only the hybrid plasmonic mode exists below a perovskite thickness of 40 nm. Adapted with permission^154^. Copyright 2018, ACS. **c** SEM image of a perovskite/Ag heterostructure. Insets: AFM image and corresponding cross-sectional profiles of a typical heterostructure. The perovskite crystals can serve as both the gain medium and the optical resonant cavity for low-threshold lasing. Adapted with permission^155^. Copyright 2016, ACS. **d** Schematic layer structure of the perovskites capped with a dielectric PMMA spacer and a Ag thin film. Not only can perovskite be protected from hydrolysis but also the lasing thresholds can be greatly reduced due to the plasmonic effect. Adapted with permission^161^. Copyright 2016, OSA. **e** Schematic of a hybrid plasmonic microcavity structure. A MAPbBr_3_ nanowire sits on a SiO_2_/Ag substrate (∼ 50 ± 1 nm) with a spacer layer of SiO_2_ (*d*_G_ ≈ 5 − 20 ± 1 nm). The exciton–photon coupling strength is enhanced by ~35% on average, which is mainly attributed to surface plasmon-induced localized excitation field redistribution. **f** Time-resolved PL spectra of MAPbBr_3_ NWs sitting on glass and SiO_2_/Ag substrates with SiO_2_ thicknesses of 20, 10, and 5 nm. Adapted with permission^162^. Copyright 2018, ACS.

NPs have been used for plasmonic–perovskite lasers. Wang et al. demonstrated plasmonic-enhanced laser emissions from all-inorganic lead-halide perovskites^153^. Uniform Al NPs were deposited onto the top surface of CsPbBr_3_ perovskites by electron beam evaporation. The resonance of the Al NPs was tuned to the emission wavelength range of CsPbBr_3_ to enhance the PL. A 20% reduction in the thresholds of whispering gallery mode lasers in CsPbBr_3_ perovskite microrods was observed. The plasmonic effects enhanced the output intensities of perovskite microlasers by more than an order of magnitude. Huang et al. demonstrated the plasmonic–perovskite laser by placing MAPb*X*_3_ perovskite nanosheets on Au patterns with a 10 nm SiO_2_ spacer (Fig. 7a)^154^. Whispering gallery modes were successfully excited by Au substrates with circle and grating patterns. Resonances within the hybrid plasmonic nanolaser could be precisely controlled by the shape and size of the bottom Au patterns instead of the top semiconductors, revealing the substrate-control concept. The standard wavelength deviation was as small as 0.3 nm. The emission wavelengths could be tuned within a 200 nm wavelength span by varying the stoichiometry of the perovskite. Hybrid plasmonic nanolasers were generated, leading to lasing even with a small perovskite thickness (<40 nm) (Fig. 7b). Li et al. used Ag NWs as nucleation centers for growing perovskites, constructing a perovskite/Ag heterostructure (Fig. 7c)^155^. Low-threshold lasing was achieved because the perovskite crystals not only were a gain medium but also formed an optical resonant cavity. The lasing modes could be modulated by tuning the plasmonic effects on the perovskite microcavities. Wu et al. placed a polymethyl methacrylate (PMMA) spacer layer and a Au NR-doped PMMA top layer on perovskite thin films^156^. The ASE threshold and intensity of the perovskite with Au NRs demonstrated an ~36% decrease and 13.9-fold increase, respectively, compared to those of perovskite films without Au NRs. The enhancement was ascribed to both the contribution of surface passivation due to PMMA and the plasmonic effects of Au NRs. Chen et al. introduced a detachable Al NP substrate to enhance MAPbBr_3_ perovskite lasing performance^157^. The lasing threshold and stimulated emission of the Al-MAPbBr_3_ hybrid system were dramatically reduced by more than 27% and improved by 10 times, respectively, due to the plasmonic effects of the Al NPs. Hsieh et al. fabricated perovskite lasers based on the configuration of Ag nanocube/CsPbBr_3_ quantum dot/Al_2_O_3_/Au. CsPbBr_3_ quantum dots were placed in the nanogap between the Ag nanocube and Au film. The threshold decreased to 1.9 W/cm^2^ at 120 K, which was due to the ultrasmall mode volume and significant Purcell enhancement at the corner of the nanocavity inside the gap^158^. Lu et al. reported upconversion lasing based on the configuration of a single MAPbBr_3_ perovskite nanocrystal (PNC)/Al_2_O_3_/TiN/silicon^159^. TiN served as a resonance-adjustable plasmonic nanocavity and generated strong localized electromagnetic fields. Both the two-photon pumping rate and emission rate were enhanced by the plasmonic resonances and enabled the upconversion lasing action. The lasing threshold decreased to 10 μJ/cm^2^, which was at least 3 orders of magnitude smaller than the threshold of the reference sample (a PNC on silicon). Huang et al. demonstrated a hybrid plasmonic surface lattice resonance (SLR) laser consisting of a MAPbBr_3_ perovskite thin film on an Ag NP array^160^. The SLR mode could be tuned to couple the green light emission of the perovskite material by the design of the NP parameters, thus enhancing the lasing performance. The pumping threshold was approximately 0.54 μW, and the linewidth of the lasing signal was only 0.28 nm.

The effects of plasmonic films on the perovskite laser performance have also been studied. Lasing performance is enhanced in lead-halide perovskites covered with PMMA and Ag (Fig. 7d)^161^. The PMMA layer can provide protection from hydrolysis in the ambient environment. The lasing threshold can be reduced by more than two orders of magnitude compared with that of bare perovskite because of the strong exciton–plasmon coupling between the Ag and perovskite films. Shang et al. reported strong exciton–photon interactions in MAPbBr_3_ perovskite NWs (Fig. 7e)^162^. Characteristic anticrossing behaviors were observed near the exciton resonance in the hybrid perovskite NW/SiO_2_/Ag cavity at room temperature, which indicated a Rabi splitting energy up to ~564 meV. The exciton–photon coupling strength was improved by ~35% on average, which was mainly ascribed to the near-field redistribution induced by the plasmonic resonances. Compared with that of the NW directly on the glass substrate, the spontaneous emission rate was enhanced by 11, 5.5, and 1.4 times with SiO_2_ thicknesses of 5, 10, and 20 nm, respectively (Fig. 7f). Yu et al. reported a plasmonic–perovskite laser by placing perovskite NWs on an Ag substrate, where an insulating spacer layer was inserted^163^. Relatively low threshold (13.5 μJ/cm^2^) operation was obtained under ambient conditions, and the devices worked well even at temperatures up to 43.6 °C. Wu et al. realized plasmonic lasing with a threshold down to ~6.5 μJ/cm^2^ at room temperature using CsPbBr_3_ NW-based plasmonic devices^164^. Polarization-sensitive pump responses and highly polarized emission parallel to the NW axis were observed due to the plasmonic modes. The Purcell effect reduced the radiative recombination lifetime of CsPbBr_3_ NWs by a factor of ~6.14. The lasing threshold of the plasmonic device increased as the NW length became larger. This may make plasmonic devices smaller and more integrated than conventional photonic devices. S. Yang et al. demonstrated an ultrafast nanolaser based on a triple-layer structure of perovskite/MgF_2_/Ag film produced with a scalable solution method^165^. The emission dynamics were accelerated by the strong plasmonic confinement of the laser modes due to the coupling between the perovskite emission and SPs. Single-mode lasing behavior was clearly observed at room temperature. The plasmonic–perovskite laser not only reduced the device size but also significantly promoted the emission efficiency. Wang et al. studied the laser performance of a single CH_3_NH_3_PbBr_3_ nanowire on a silver film without any insulating layer^166^. The nonhybridized plasmonic nanowire lasers exhibited ultrafast lasing dynamics with an approximately 1.9 ps decay rate and a 1 ps peak response time. The low threshold was due to the ultraflat single-crystal silver films and high-quality single-crystal perovskite nanowires.

Plasmonic–perovskite lasers show great potential for use in integrated optics due to their small size, low threshold, and controlled wavelength and polarization. The Purcell effect can be enhanced by plasmonic resonances, which increases the light–matter interaction and emission intensity while reducing the emission lifetime, thus enabling faster lasers and faster LEDs. Future challenges include the ability to achieve efficient carrier injection and the realization of an electrically pumped plasmonic–perovskite laser. In addition, smarter nanostructures and plasmonic–perovskite interface designs are needed to further reduce the device size and footprint, as well as reduce the lasing threshold.

## Plasmonic–perovskite sensors

### Plasmonic–perovskite photodetectors

**Fig. 8:**
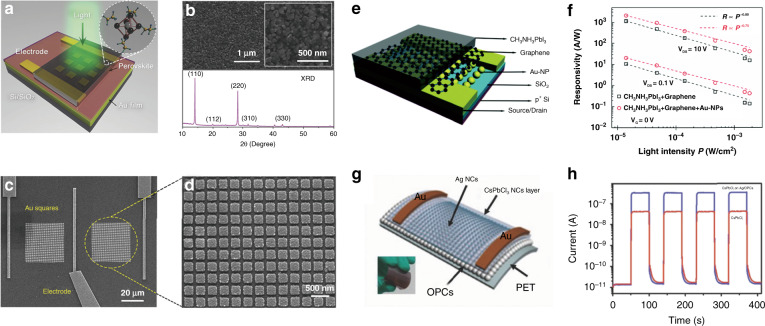
Plasmonic–perovskite photodetectors. **a**–**d** Organic-inorganic hybrid perovskite PD on arrays of Au nanostructures. The maximum EQE of this PD is as high as approximately 65%, 2.5 times that on a usual Si/SiO_2_ substrate without Au nanostructures, which is due to the large, localized *E*-field induced by the SPR. **a** Schematic of the device on a plasmonic substrate. **b** SEM images of the plasmonic substrate. **c** The dependence of the photocurrent on the incident laser power for the device on Si/SiO_2_ and plasmonic substrates at zero bias. **d** EQE of devices on Si/SiO_2_ and plasmonic substrates. Adapted with permission^168^. Copyright 2019, Wiley. **e**, **f** Graphene/MAPbI_3_ hybrid PDs integrated with Au NPs. **e** Schematic of the Au NP/graphene/CH_3_NH_3_PbI_3_ hybrid PDs. **f** Responsivities versus light intensity at different *V*_DS_ biases. The dependencies of the responsivities on the light intensity are fitted with power functions with the listed fitting parameters. Compared with pristine graphene-MAPbI_3_ devices, this device has two times higher photoresponsivity and a faster photoresponse speed. This enhancement can be attributed to the improved light absorption in the perovskite layer due to the plasmonic effect of the Au NPs. Adapted with permission^170^. Copyright 2016, RSC. **g**, **h** CsPbCl_3_/Ag/OPC hybrid PDs. **(g)** Schematic of CsPbCl_3_ on Ag/OPC PDs. **h** On-off switching properties measured under 365 nm light illumination (10 mW/cm^2^) at a bias of 3 V. The photocurrent of CsPbCl_3_ PD with Ag/OPC is 682% higher than that of CsPbCl_3_ PD without Ag/OPC. Adapted with permission^176^. Copyright 2018, Wiley.

The performance of PDs can be improved by plasmonic nanostructures because they enhance the coupling between incident light and the semiconducting materials that generate photocurrents. In the work of Dong et al., Au NCs were incorporated into CsPbBr_3_ NC visible-light PDs^167^. The PDs with Au NCs exhibited a higher sensitivity with an on/off ratio that was more than one order of magnitude larger than that of the initial device at a 2 V bias under 532 nm laser illumination (4.65 mW/cm^2^). Moreover, the photocurrent increased by 238% (from the original 245.6 μA to 831.1 μA) with the incorporation of Au NCs due to the near-field plasmonic enhancement. B. Du et al. fabricated an organic-inorganic hybrid perovskite PD with periodic Au nanosquares (Fig. 8a–d)^168^. The maximum EQE of this PD was up to 65%, which was 2.5-fold higher than that of a similar hybrid perovskite placed on a usual Si/SiO_2_ substrate without Au nanosquares. The large, localized *E*-field induced by the SPR of Au nanostructures was responsible for the enhancement. With a configuration similar to that in Fig. 8a, Gu et al. studied the effect of Au nanotriangles on PD performance^169^. The EQE of MAPbI_3_-Au was ~3 times higher than that of the MAPbI_3_ film without Au nanotriangles. The photoresponsivity of the MAPbI_3_-Au-based PD was 51 mA/W at 10 V with a power density of 1.5 W/cm^2^ and an EQE of 12.6% for an illumination wavelength of 450 nm. Au NPs were integrated into the graphene/MAPbI_3_ hybrid PDs (Fig. 8e, f)^170^. The device with Au NPs exhibited a 2-fold higher photoresponsivity and a faster photoresponse speed compared with those of pristine graphene-MAPbI_3_ devices. Plasmon-enhanced light harvesting was improved on the perovskite–graphene interface, which led to a higher PD operation speed and carrier extraction efficiency than devices without Au NPs. Ghosh et al. reported an air-processed high-performance self-powered hybrid perovskite PD whose ETL was embedded with plasmonic Ag NPs^171^. At zero bias, the ETL-free Ag NP-perovskite hybrid device showed a ∼45% enhanced responsivity and operated with a ∼3 times faster photoresponse than the pristine device. The enhancements were due to a series of positive effects introduced by the plasmonic Ag NPs, such as enhanced light absorption, hot electron generation, and improved charge extraction and transport.

Ji et al. showed a quasi-2D perovskite PD with SiO_2_-coated AuAg-alloyed nanoprisms (AuAg-NPrisms@SiO_2_) between the PEDOT:PSS and perovskite layers^172^. The PDs with AuAg-NPrisms@SiO_2_ exhibited a high EQE of 1670% at a low driving voltage of −0.3 V. Their responsivity and detectivity reached 7.15 A/W and 3.2 × 10^13^ Jones, which were 51.2% and 68.4% higher than those of PDs without NPs. The enhancement was because the AuAg-NPrisms@SiO_2_ led to high-quality crystalline quasi-2D perovskite films and stronger light absorption in the active layer, accelerating exciton dissociation at the interface and facilitating charge transport. Li et al. reported a PD composed of perovskite/Al NP/anodic aluminum oxide (AAO)^173^. The hybrid plasmonic–perovskite nanostructures showed a 43-fold increase in the photocurrent, which was due to the enhanced *E*-fields around the Au NPs. Wang et al. used plasmonic bowtie nanoantenna arrays to boost the performance of a perovskite PD^174^. The photoresponse and detectivity of the plasmonic–perovskite PD increased by ~2962% and more than 30 times compared with those of a Si/SiO_2_-based conventional PD, respectively. Electric fields were greatly enhanced around the bowtie nanoantenna arrays due to the LSPR effect, which led to the improvement. La et al. realized a perovskite PD by embedding assembled Au NPs in the PEDOT:PSS layer^175^. Charge extraction/transport and optical absorption were enhanced by the assembled Au NPs due to the good conductivity and plasmonic resonance of the Au NPs. The responsivity, noise equivalent power (NEP), and response speed of the perovskite with Au NPs were ~2063 A/W, ~1.93 × 10^–14^ W/Hz^1/2^, and ~300 ns, respectively, which were approximately 4.1-, 15.6-, and 2.8-fold increases, respectively, compared with those of a perovskite PD without NPs.

Ag films composed of Ag NCs and CsPbCl_3_ NCs were used for high-performance flexible ultraviolet photodetectors (Fig. 8g, h)^176^. The luminescent intensity enhancement of the CsPbCl_3_ NCs showed a more than 150-fold improvement with an estimated emission efficiency of 51.5%. The improvement was due to the combined excitation and emission field enhancement. The CsPbCl_3_/Ag/opal photonic crystal (OPC) hybrids exhibited a dark current of 10^–11^ A, detectivity of 9 × 10^14^ Jones, response time of 28 ms, and response linewidth of 30 nm. This performance was claimed to exceed that of commercial silicon PDs at the time that the paper was published.

By integrating plasmonic nanostructures with perovskites, enhanced light absorption can be attained, which opens the door for high-performance photodetectors. Moreover, plasmonic metals can also act as electrodes and perform carrier extraction without increasing the complexity of the configuration. The challenge will be to avoid carrier tunneling between metals and perovskite and simultaneously increase the conductivity. Different combinations of halide perovskite materials and plasmonic nanostructures should be explored to maximize photodetector performance across the desired wavelength span.

### Plasmonic–perovskite optical sensors

Recently, halide perovskites have been explored for applications as refractometric sensors, surface-enhanced Raman scattering (SERS), and biologic sensors. Elshorbagy et al. theoretically reported a perovskite refractometric sensor with an extruded array of high aspect-ratio dielectric pyramids, which excited SPRs at its front surface by grating coupling with the metal surface^177^. The generated photocurrent acted as the sensing signal, where selective absorption with a spectral response narrower than 1 nm was achieved. Without relying on the spectral acquisition scheme, the perovskite device refractometric sensor used an optoelectronic interrogation method. The device showed considerable sensing performance in the refraction index range between 1.0 and 1.1. The figure of merit (FOM) could reach 1005 RIU^−1^. In addition, the perovskite refractometric sensor also possessed the advantages of a simple signal acquisition procedure and low cost.

**Fig. 9:**
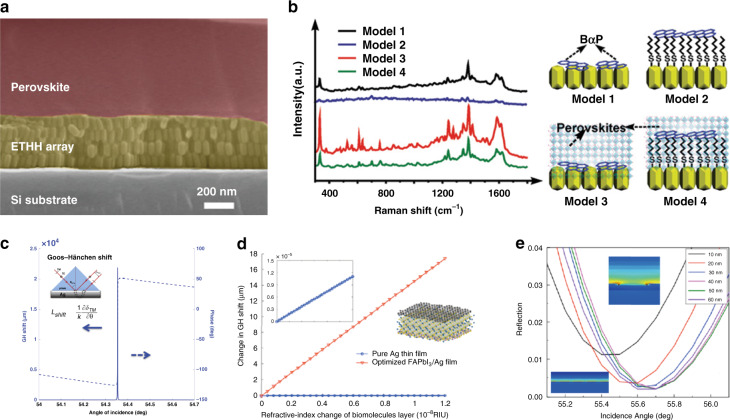
Plasmonic–perovskite optical sensors. **a**, **b** Superficial-layer-enhanced Raman scattering (SLERS) technique based on covering elongated tetrahexahedral (ETHH) Au NP arrays with a CH_3_NH_3_PbBr_3_ film. Noninteracting analytes, such as benzo[α]pyrene (BαP) that has chronic toxicity and can easily adhere to human skin or muscles to cause carcinogenesis, is detected. **a** Cross-sectional SEM images of the perovskite film on ETHH NP arrays. **b** Raman spectra of BαP in four different models without (Model 1 and Model 2) and with (Model 3 and Model 4) the perovskite. BαP is segregated from the ETHH arrays by n-alkyl thiols in Model 2 and Model 4. The Raman signals are significantly enhanced with the perovskite because the perovskite film, as a dielectric medium, slows the attenuation of the electromagnetic evanescent wave and the LSPR decay is suppressed along the vertical direction away from the nanostructured surface. Adapted with permission^178^. Copyright 2019, Wiley. **c** Optical phase and GH shift as a function of the angle of incidence near the SPP resonance angle of 54.36°. The sensing substrate consists of a bilayer FAPbI_3_ on a 45-nm silver thin film. **d** Simulations showing that a plasmonic sensor based on graphene/FAPbI_3_/hBN/Ag can detect small refractive index changes on the order of 10^–8^ RIU, which is 4 orders of magnitude more sensitive than plasmonic sensors based on a simple Au substrate. The presence of 2D perovskite layers of a suitable thickness can lead to deeper SPP resonance dips, which correspond to a sharper optical phase change and a larger GH shift. **e** Simulated angular reflectance spectra for a device consisting of bilayer FAPbI_3_ on silver nanogrooves with various groove widths. The insets show that the enhanced near fields lead to deeper spectral dips. Adapted with permission^214^. Copyright 2020, MPDI.

Qiao et al. demonstrated a superficial-layer-enhanced Raman scattering (SLERS) technique for detecting species that were noninteractive with Raman substrates. The configuration consisted of elongated tetrahexahedral Au NP (ETHH Au NP) arrays covered with a superficial CH_3_NH_3_PbBr_3_ film (Fig. 9a, b)^178^. These ETHH NP arrays provided uniform and high-density SERS hotspots, and the perovskite film acted as a dielectric media to slow the attenuation of the electromagnetic evanescent wave of the hotspots, which made SLERS occur in the superficial layer rather than just on the surface of the ETHH Au NPs. Zeng et al. presented a sensor based on a hybrid metasurface consisting of 2D perovskites and metal thin films^179^. The device showed a sharp optical phase change and a Goos-Hänchen shift around the wavelength of the SPP (Fig. 9c). The Goos-Hänchen (GH) shift exhibited a sensitivity of 900,000 μm/RIU, which was more than 4 orders of magnitude higher than that of a pure Ag sensing substrate (800 μm/RIU) (Fig. 9d). When silver nanogrooves were used (Fig. 9e), coupling between the LSPR and SPP waves led to a deeper dip in the angular spectra, implying a further improvement in the detection sensitivity. Such 2D perovskite-based metasurfaces show promise for use as ultrasensitive in situ biosensors.

Halide perovskites have not been widely studied as sensors; thus, reports of plasmonic–perovskite sensors are rarer. Nevertheless, plasmonic sensors are a mature technology with numerous chemical, biological and medical detection applications. The combination of plasmonics and perovskite technologies will break new boundaries in sensitivity and pave the way for a truly compact and integrated optical sensor platform. We also envision the use of plasmonic–perovskite sensors to detect other physical and environmental parameters in different technological domains.

## Other plasmonic–perovskite applications

**Fig. 10:**
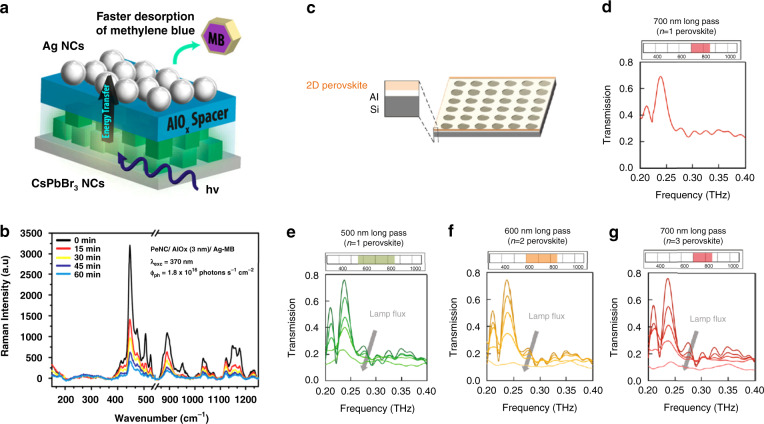
Plasmonic perovskite for driving chemical reactions and the photoinduced modulation of THz resonances. **a** Schematic of the proposed perovskite NC/AlO_x_/Ag NC antenna for driving chemical reactions by coupling them to plasmonic Ag NCs, where CsPbBr_3_ NCs are used as light-harvesting energy-transfer donors, while Ag NCs are employed as acceptors and catalysts. A directional funneling of energy from the perovskite toward the silver is established to drive dye desorption. **b** Representative photochemical reaction experiment showing the decrease in the Raman signal of the PeNC/AlO_*x*_ (3 nm)/Ag-MB sample under illumination. The decrease in the Raman signals points to the desorption of MB dye from the Ag NC surface induced by plasmon excitation through energy transfer from the PeNCs. Adapted with permission^181^. Copyright 2019, ACS. **c**, **d** Photoinduced modulation of the THz plasmonic resonances by spin coating an MAPbI_3_ perovskite on top of metallic hole arrays, which in turn are fabricated on a silicon substrate. **c** Schematic diagram of the device composed of a 2D perovskite thin film/Al nanohole array/high-resistivity silicon substrate. **d** Transmission spectrum of the nanohole array demonstrating negligible attenuation for the *n* = 1 perovskite/Si sample when excited with a 700 nm long-pass filter (i.e., no excitons are generated). **e**–**g** Optical excitation-induced attenuation of the transmitted THz transmission through the Al nanohole array. These arrays were excited using 500 nm long-pass, 600 nm long-pass, and 700 nm long-pass filters. The optical excitation range is shown above each spectrum. Nearly 100% attenuation of the plasmonic resonances is achieved as the lamp flux is increased (the arrow direction corresponds to increasing lamp flux). Adapted with permission^182^. Copyright 2017, Springer Nature.

The performance of perovskites in photocatalysis can also be enhanced by plasmonic NPs. Ag NPs have been synthesized on perovskite orthorhombic KNbO_3_ NWs by facile photoreduction^180^. Ag/KNbO_3_ nanocomposites with different Ag contents (0.4–2.8 wt%) have been used to monitor the degradation of aqueous rhodamine B under UV and visible light. As the Ag content is increased, the UV-induced photoreactivity first increases, reaching a maximum at a Ag content of ~1.7 wt%, and then decreases. The maximum photoreactivity is ~13-fold higher than that of pristine KNbO_3_ without Ag NPs. In contrast, visible light-induced photoreactivity shows a monotonic positive correlation with Ag contents from 0.4 wt% to 2.8 wt%. Saris et al. used CsPbBr_3_ NC/AlO_*x*_ composite films with Ag NCs to drive chemical reactions based on the coupling between perovskite and Ag NCs (Fig. 10a, b)^181^. It was found that energy migrated from the perovskite NCs toward the Ag NCs. This energy migration was utilized to boost plasmon-mediated methylene blue desorption, in which coupling to the perovskite NCs enhanced the spatial and spectral absorption of the chemical reaction. Chanana et al. proposed a triple-layered structure of (C_6_H_5_C_2_H_4_NH_3_)_2_(CH_3_NH_3_)_n−1_(PbI_4_)_n_/MAPbI_3_/Al hole arrays/silicon substrate (Fig. 10c) for the photoinduced modulation of THz resonance^182^. The sample with *n* = 1 showed a distinct THz transmission peak, exhibiting no optical intensity dependence when illuminated at 700 nm (Fig. 10d), since that wavelength did not correspond to photogenerated excitons in the perovskite layer. When the samples were illuminated in the wavelength range where excitons were formed, the increased THz absorption within the holes led to lower overall THz transmission with increasing optical intensity (Fig. 10e–g). This work indicated that the plasmonic effect could be tuned by the perovskite. Zhou et al. reported a polarization-dependent modulation based on hybrid perovskite plasmon-induced transparency (PIT)^183^. A MAPbI_3_ film was coated on the PIT acting as a photoactive medium. Even with ultralow laser fluence pumping, a significant reduction in PTI transmission was observed for the two polarizations at 0.86 and 1.12 THz with a recovery time of 561 ps, demonstrating the high sensitivity and ultrafast modulation speed of the device.

## Conclusions and outlook

In recent years, researchers have demonstrated the use of plasmonics to enhance the performance of perovskite-based solar cells, light emitters, photodetectors, sensors, and many other applications. Regarding plasmonic PSCs, the majority of studies are based on NPs because NPs can be synthesized and easily incorporated into devices. We observed several trends from the vast amount of literature on NP-assisted PSCs that we examined: (1) the PCE of PSCs can be significantly enhanced by plasmonic NPs; (2) PSCs with plasmonic NPs incorporated at the perovskite-m-TiO_2_ interface or simultaneously at multiple positions can potentially achieve the largest PCE enhancement; (3) NPs with a core–shell structure, shape modification, and dimer show stronger effects than that of simple spherical NPs; (4) alloyed NPs show stronger enhancement than that of a single material; and (5) the size and concentration have different effects on the current, voltage, and FF; thus, they need to be optimized carefully. Based on these observations, we infer that alloyed core–shell nanorods with sharp tips and sub-10 nm gaps positioned at the perovskite-m-TiO_2_ interface are promising for substantially increasing PV performance. The size and concentration may be optimized when <50 nm and <2 wt%, respectively. Plasmonic (nanostructured) films have not been widely used in PSCs because their fabrication is usually more complicated, and they can introduce more loss. Nevertheless, plasmonic films can still be promising for high PV performance, as plasmonic films provide a larger penetration depth of SPPs, which can exert a larger influence on *E*-field enhancement, conductivity, and other factors. In addition to increasing light absorption and photocurrent generation, plasmonic NPs and films can also enhance the light emission of perovskites, which benefits the applications of LEDs and lasers. The Purcell effect can be enhanced by plasmonic resonances, which increase the light–matter interaction and emission intensity while reducing the emission lifetime, thus enabling faster LEDs and lasers. Plasmonic NPs and films also significantly improve the performance of perovskites used for optical detection and sensing, photocatalysis, and THz modulation. Simulations and experiments demonstrate that these enhancements are mainly attributed to the plasmonic-optical (near-field absorption and far-field scattering) effect.

Although the combination of perovskite and plasmonics has progressed rapidly, there are still some challenges to overcome. First, the quality, stability, and reliability of halide perovskites remain major problems for perovskite-related applications. In addition to efforts to improve the intrinsic properties of perovskites, it would be interesting to study changes in the quality, stability, and reliability after the addition of plasmonic nanostructures in the vicinity of perovskites, especially the use of NPs to improve these properties. Second, novel plasmonic nanostructures can be further explored and optimized to meet specific application demands^184–186^, through the control of the wavelength, light angles and polarizations, optical confinement, hotspot positions, coupling and proximity effects. Perovskite materials and compositions that can seamlessly integrate with plasmonic nanostructures can be identified based on realistic fabrication conditions and constraints, band alignment, etc.^12,36,187^. Light manipulation in plasmonic nanostructures and adaptive perovskite materials will lead to more applications and the further maximization of their performance. Third, the mechanism behind plasmonic–perovskite interactions should be studied in greater depth. Although macroscopic enhancement mechanisms are well understood, other intricate phenomena (such as optical quenching, carrier tunneling, and quantum effects) at the nanoscale remains unanswered. In addition, the separation of the various optical and electrical effects is still an open question along with determining the exact contribution from each effect. Fourth, machine learning (ML) is a powerful tool for designing and screening perovskite materials^188,189^, as well as for inverse design in nanophotonics^190–192^. There are many opportunities to apply ML toward plasmonic perovskites, such as to assist in the determination of the material, structure, and device architecture of plasmonic–perovskite systems for improved performance. All these challenges and opportunities encourage deeper theoretical analysis and further experimental studies in the years to come.
